# Recent Advances in High‐Entropy Nanozymes: From Fabrication to Applications

**DOI:** 10.1002/advs.78335

**Published:** 2026-11-10

**Authors:** Yongjian Ai, Weibo Zhang, Pengjie Wang, Wentao Xu, Huiyuan Guo, Hong‐bin Sun, Meng‐Qi He, Fazheng Ren, Qionglin Liang

**Affiliations:** ^1^ MOE Key Laboratory of Precision Nutrition and Food Quality Department of Nutrition and Health China Agricultural University Beijing People's Republic of China; ^2^ Department of Chemistry College of Science Northeastern University Shenyang People's Republic of China; ^3^ State Key Laboratory of Respiratory Health and Multimorbidity Institute of Basic Medical Sciences Chinese Academy of Medical Sciences School of Basic Medicine Peking Union Medical College Beijing People's Republic of China; ^4^ Beijing Key Laboratory of Digital Intelligent Organoids and Organ‐on‐a‐Chip MOE Key Laboratory of Bioorganic Phosphorus Chemistry & Chemical Biology State Key Laboratory of Flexible Electronics Technology Department of Chemistry Tsinghua University Beijing People's Republic of China

**Keywords:** catalytic mechanisms, high‐entropy nanozymes, multifunctional applications, nanozymes, synthetic methodology

## Abstract

Nanozymes are artificial enzymes designed to circumvent the inherent limitations of natural enzymes. They have emerged as a transformative paradigm in biomimetic catalysis. High‐entropy nanozymes (HEAzymes) are nanomaterials that consist of high‐entropy alloys, oxides, sulfides, and other high‑entropy compounds that exhibit enzyme‐like catalytic activity. HEAzymes have recently garnered substantial attention as a next‐generation nanozyme. The rapid evolution of this field has been propelled by synergistic advances in nanotechnology, biotechnology, catalysis, and computational modeling, offering unprecedented opportunities for rational design and broad‐spectrum applications. This review provides a comprehensive and timely overview of state‐of‐the‐art developments in HEAzymes, systematically spanning synthetic methodologies, catalytic mechanisms, and multifunctional applications. We classify fabrication strategies into thermodynamically driven and kinetically controlled routes, elucidate enzyme‐mimetic catalytic behaviors and their underlying mechanistic principles, and critically examine recent advances in antioxidative, therapeutic, biosensing, energy‐related, and emerging applications. Finally, we discuss prevailing challenges and outline prospective directions to guide future research. By spotlighting the frontier of HEAzyme research, this review aims to foster interdisciplinary collaboration and catalyze continued innovation in nanozyme science.

## Graphical abstract

To highlight key achievements and clarify the current landscape of high‐entropy nanozymes (HEAzymes), this review summarizes state‐of‐the‐art developments ranging from thermodynamically and kinetically controlled synthetic strategies; enzyme‐like catalytic types, catalytic characteristics, and underlying mechanisms; to their antioxidative, therapeutic, biosensing, energy‐related, and other emerging applications. We think this review can spotlight the HEAzyme research frontier, inspire multidisciplinary researchers, and accelerate nanozymes development.

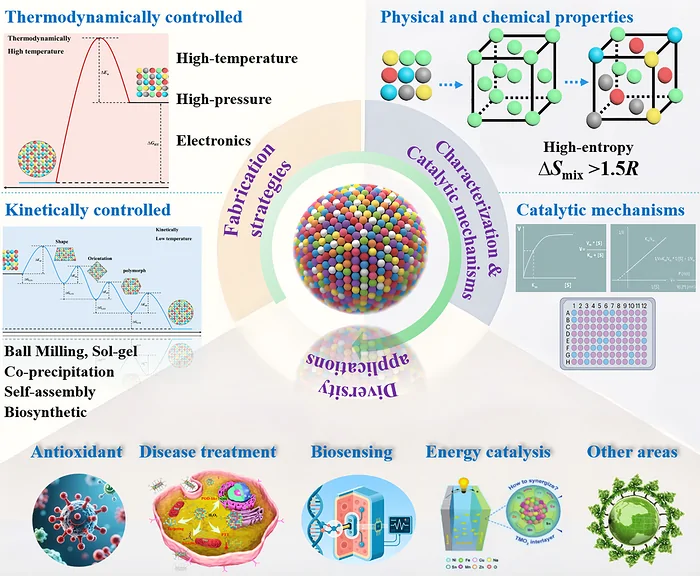

## 1 Introduction

Nanozymes are a class of artificial enzymes that integrate the distinctive physicochemical properties of nanomaterials with catalytic activities analogous to natural enzymes [1, 2, 3, 4, 5, 6]. In 2007, Yan's group first reported that Fe_3_O_4_ nanoparticles (NPs) possess horseradish peroxidase (HRP)‐like activity, thereby establishing the concept of nanozyme [7]. In 2020, the publication of nanozymology formally recognized nanozyme as an independent interdisciplinary research field [8, 9, 10]. Compared with natural enzymes, nanozymes offer several advantages of highly tailorable structures, facile large‐scale synthesis, low cost, ease of surface modification, high stability against heat, acid, and alkali, and catalytic activity that external stimuli can modulate [11, 12, 13]. Benefiting from the rapid development of nanotechnology, analytical chemistry, and theoretical computation, a broad spectrum of nanozymes mimicking peroxidase (POD) [14], catalase (CAT) [15], superoxide dismutase (SOD) [16], glutathione peroxidase (GPx) [17], glucose oxidase (GOD), phosphatase, and other nanozymes have been successfully constructed [18, 19, 20]. These nanozymes are regarded as powerful complements and even potential replacements for natural enzymes in various applications [21]. In recent years, metal‐based, non‐metal‐based, and their composite nanomaterials have all been extensively explored for nanozyme construction. The emergence of nanozymes has blurred the traditional boundary between organic and inorganic catalysts [22, 23, 24]. Recognizing their transformative potential, the International Union of Pure and Applied Chemistry (IUPAC) selected nanozymes as one of the top 10 emerging technologies of 2022. At the same time, the Chinese Academy of Sciences included nanozyme research in the top 10 chemistry and materials‐science frontiers. The World Economic Forum highlighted nanozymes among the 2025 top 10 emerging technologies [25, 26, 27, 28]. Nanozyme research has entered an era of deep interdisciplinary integration, focusing on theragnostic approaches for major diseases of tumors, cerebral infarction, neurodegenerative disorders, anti‐bacterial/anti‐inflammatory applications, disease prevention and control, specific biomarker detection, antioxidant effects, anti‐drug‐resistant bacteria, and environmental monitoring and remediation [29, 30, 31]. Many efforts were deepening fundamental investigations, create new materials to expand the nanozyme library and boost activity, and align with critical needs in precision medicine, accurate diagnosis, and environmental governance to accelerate from basic science to industrialization. Nevertheless, this research field is still in its infancy. Limited enzyme‐like types, modest enzyme‐like catalytic efficiency, poorly understood mechanisms, insufficient specificity/selectivity, and lack of targeting ability collectively hinder clinical translation [32, 33].

In recent years, a growing variety of advanced materials have been rationally designed and synthesized to fabricate nanozymes [34, 35, 36, 37]. The compositional landscape has progressed from conventional nanomaterials, through ultrafine NPs [38] and sub‐nanometer clusters [39], to the current frontier of single‐atom catalysts [40]. Since 2004 Yeh and Cantor et al. manufactured multicomponent alloys near‐equal atomic proportions and put forward the concept of high‐entropy alloys (HEAs), this theme has attracted much attention [41, 42, 43, 44]. High‐entropy materials (HEMs) have emerged as a particularly exciting platform that positions high‐entropy nanozymes (HEAzymes) at the forefront of cutting‐edge research; these HEAzymes comprise HEAs, oxides, sulfides, and other high‐entropy compounds (HECs) exhibiting enzyme‐like activity [45, 46, 47]. The HEAs mean the solid solutions containing five or more principal metals in near‐equimolar ratios [48, 49, 50, 51, 52]. Their possess four hallmark effects of high configurational entropy, severe lattice distortion, sluggish diffusion, and cocktail‐like properties [53, 54, 55]. These characteristics yield low overpotentials, high stability, and rapid reaction kinetics [56]. Multielement synergy suppresses brittle intermetallic phases, stabilizing single‐phase solid solutions and enabling precise tailoring of electronic/geometric structures for optimal catalytic performance [57]. It is obviously that metals such as Fe, Co, Ni, Cu, Zn, V, Mn, Mo, Ru, Rh, Pd, Ag, Ce, Pt, and Au possess vacant d or f orbitals that can form low energy barrier transition states with substrates, thereby markedly lowering activation energies [58, 59, 60, 61]. Furthermore, multielement d‐orbital coupling in HEAzymes significantly enhances electron transfer efficiency, allowing the mimicry different enzymatic‐like activities [62, 63, 64]. The high‐entropy framework also affords exceptional stability and establishes a versatile platform for composition‐dependent activity tuning [65, 66]. The above lead HEAzymes have great potential in tumor immunotherapy, anti‐inflammatory, pathogen and biomarker detection, and pollutant degradation [67, 68, 69].

Herein, to provide researchers in materials, chemistry, biology, nanotechnology, pharmacy, and medicine with an overview of HEAzymes, this review systematically summarizes their thermodynamically driven and kinetically controlled synthesis based thermodynamically and kinetically controlled methods, determination and enzyme‐like catalytic mechanisms characterization, and diverse applications in antioxidation, disease treatment, biosensing, and energy catalysis (Scheme 1). We first detail the choice of synthetic strategies based on high‐temperature, high‐pressure, electronics synthesis, ball milling, co‐precipitation, sol‐gel and self‐assembly preparation methods with different metallic precursors. Then, we outline the catalytic characteristics and mechanisms of distinct nanozyme types. Following, we review the latest progress in antioxidative, therapeutic, biosensing, and energy‐related applications. Finally, we highlight current challenges and propose future directions for HEAzymes research. We anticipate that this review can accelerate the development and translation of next‐generation high‐performance nanozymes.

**SCHEME 1:**
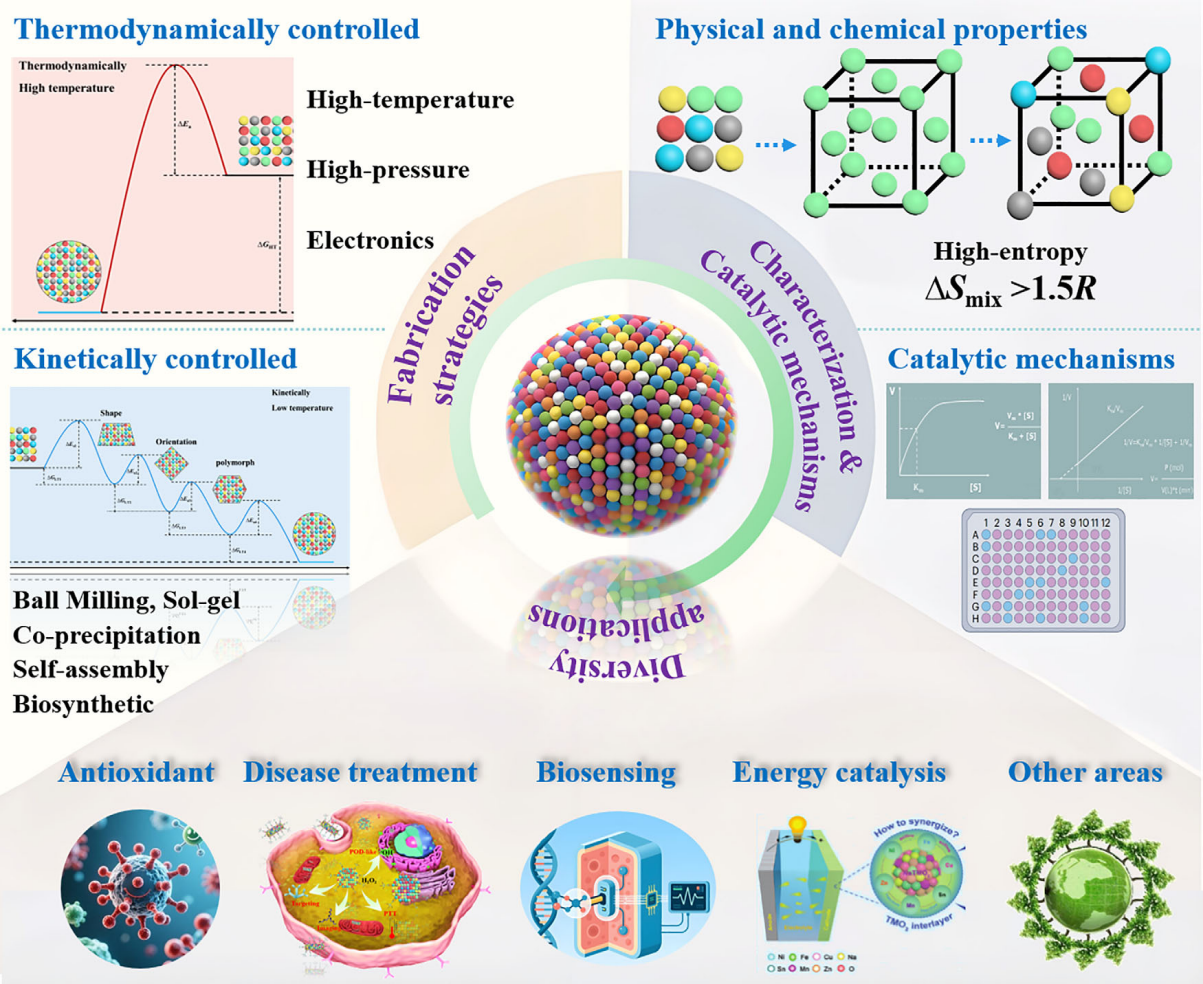
The overview of HEAzymes: from fabrication strategy and determination to versatile applications.

## 2 Synthetic strategies for HEAzymes

As shown in Figure 1, the development of HEAzymes can be traced back to 2004 when the concept of HEMs first proposed. Following, high‐entropy oxides (HEOs) subsequently emerging in the catalysis field in 2015 [70]. A groundbreaking breakthrough occurred in 2018 when Hu and colleagues pioneered the synthesis of octonary HEA NPs via a high‐temperature thermal shock technique [71]. This work was followed by systematic expansion to decenary HEO NPs for oxygen reduction reactions in 2021 [72] and fifteen elements alloy NPs with observed local strain and lattice distortion in 2023, alongside the establishment of a comprehensive theoretical framework elucidating synthesis‐structure‐property relationships and data‐driven discovery [53]. In 2023, our group reporting the first application of ultrasmall HEA NPs with enzyme‐like activity for enhanced tumor photothermal therapy [45]. HEAzymes are a class of nanomaterials that consist of HEAs, oxides, sulfides, and other high‑entropy compounds, and they exhibit enzyme‐like catalytic activity. During 2024–2025, this domain witnessed rapid expansion with clinically oriented innovations. These developments demonstrate the unique advantages of HEAzymes in synergistic enhancing catalytic activity, selectivity, and stability. HEAzymes acted as a transformative paradigm in nanomedicine and healthcare [73, 74, 75, 76, 77]. In the same year, advanced characterization techniques of atomic electron tomography have enabled atomic‐resolution imaging of elemental distributions and revealed atomic coordination environments and electronic structures [78]. This work confirmed the single‐phase solid solution structure and homogeneous multielement mixing characteristic of HEA NPs.

**FIGURE 1:**
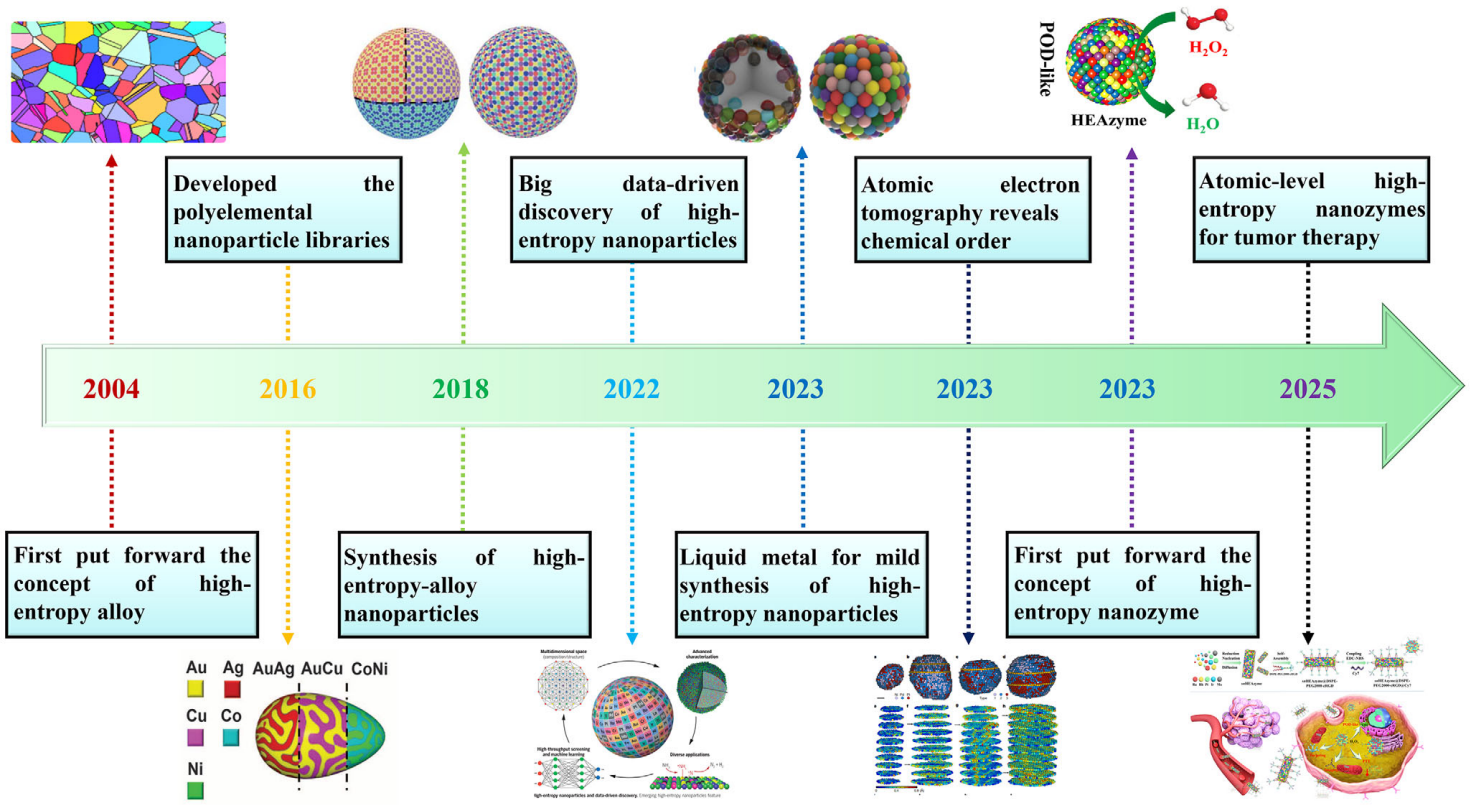
The development history of HEAzymes.

As a new class of multicomponent alloys under near‐equiatomic conditions, HEAs breaking through the traditional paradigm of alloy design centered on a single principal element [79, 80, 81]. It was widely believed that such a chemically complex environment would readily lead to the formation of multiple brittle intermetallic compounds. However, many experiments revealed that these alloys form a single‐phase solid‐solution structure, with multiple principal elements occupying lattice sites in an almost random manner (Figure 2A). Follow‐up studies confirmed that their phase stability stems mainly from the significant increase in configurational entropy during mixing, which is sufficient to overcome the loss in mixing entropy [82, 83, 84]. Although binary and ternary concentrated solid solutions had been reported earlier, the successful synthesis of such multi principal element systems shifted the research focus from the edge regions of phase diagrams toward the central region of multicomponent phase diagrams dimensional composition space. Consequently, HEAs were clearly defined as alloy 5% and 35% [85, 86, 87]. These materials not only retain traditional advantages like high hardness and wear resistance, but also exhibit a good synergy between strength and ductility at low temperatures, along with excellent fracture resistance [88, 89, 90, 91, 92]. This theoretical framework was first proposed by Yeh and his collaborators have now gained wide verification and recognition [93]. Subsequently, researchers successively synthesized a series of HEAs with simple crystal structures such as face‐centered cubic (fcc), body‐centered cubic (bcc), and hexagonal close‐packed (hcp), and systematically studied them [94]. The design concept based on entropy stabilization later rapidly expanded beyond metallic systems, giving rise to various HECs, including high‐entropy ceramics [95], high‐entropy organic framework materials [96], and high‐entropy halide perovskites [97], forming a broader research field. Through precise control of composition, configuration, and stoichiometry, HEMs have demonstrated great potential in many frontier areas such as catalysis energy storage and conversion, magnet and electronics, substantially expanding the compositional space and the scope for performance tuning in material design [98, 99, 100, 101]. However, the controllable synthesis of HEMs faces severe challenges due to their extremely high chemical complexity [102, 103, 104, 105]. In this section, we reviewed the recent advances of synthetic strategies based on different metallic precursors, namely the thermodynamically controlled and kinetically controlled synthesis methods (Figure 2B and Table 1). High‐temperature non‐equilibrium synthesis usually drives the system directly toward the thermodynamic equilibrium state. Whereas low‐temperature solution synthesis provides another powerful tool that enables precise control over composition and morphology at nanoscale [106, 107, 108, 109]. The solution environment can effectively circumvent the diffusion limitations encountered in solid‐state synthesis. Although the non‐equilibrium process in solution may trap the system in multiple metastable states, it also offers more flexibility for microstructural engineering, and the kinetic barriers in stages such as nucleation and growth are relatively low, facilitating high‐precision control over structural parameters such as size, phase state and orientation [110, 111, 112, 113].

**FIGURE 2:**
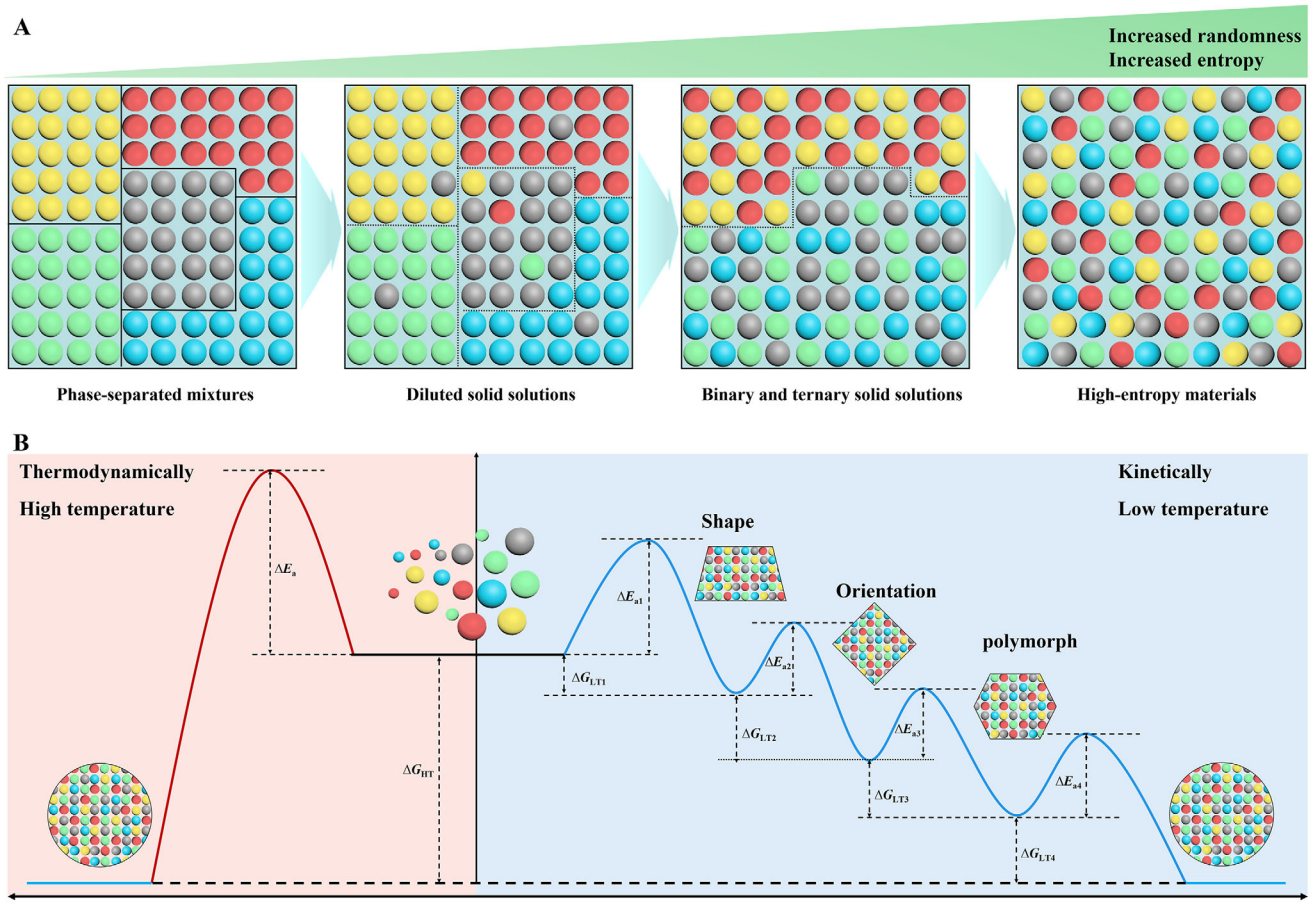
Design and synthesis principles of HEA NPs: (A) synthesis methodologies and the evolution of structural disorder and configurational entropy; (B) thermodynamic and kinetic processing routes and associated energy landscapes.

**TABLE 1:** Comparison of thermodynamically controlled and kinetically controlled synthesis routes for HEAzyme fabrication.

Comparison dimension	Thermodynamically‐controlled	Kinetically‐controlled
Crystallinity	Generally high; favors thermodynamically stable phases	Generally lower; may yield metastable or amorphous phases
Particle size	Relatively large; sufficient time for growth	Relatively small; limited by reaction rates
Elemental homogeneity	Typically excellent; diffusion reaches equilibrium	Often compromised; elemental distribution constrained by kinetics
Phase purity	High; tends to produce a single stable phase	Lower; prone to multiphase coexistence or metastable phases
Scalability	Broader process window; good batch‐to‐batch consistency	Highly sensitive to process parameters; scale‐up is challenging
Equipment requirements	Typically requires high temperatures/prolonged annealing; higher energy input	Milder reaction conditions; demands precise control systems
Cost efficiency	High energy cost, but relatively simple post‐processing	Lower energy cost, yet requires stringent process control
Energy consumption	High‐usually necessitates elevated temperatures and long durations	Low‐operates under milder conditions
Phase/compositional scope	Restricted to thermodynamically stable phases; limits compositional space	Capable of trapping metastable phases; expands compositional space
Reproducibility	Good; equilibrium states are less sensitive to initial conditions	Poor‐highly sensitive to temperature, time, precursor addition rate, etc.
Synthesis duration	Hours to days	Minutes
Post‐processing complexity	Relatively straightforward	Require additional steps to stabilize metastable products
Applications	HEAzymes demanding high crystallinity, high phase purity, and uniform elemental distribution	HEAzymes targeting nanoscale dimensions, metastable phases, rapid synthesis, or mild fabrication conditions

### 2.1 Thermodynamically Controlled Synthesis Methods

Thermodynamically controlled synthesis aims to drive the reaction system toward the equilibrium state with the lowest free energy, enabling the formation of highly stable HEAzymes structures. This approach typically yields single‐phase solid solutions characterized by high crystallinity, low defect density, and well‐defined equilibrium morphologies [114]. These structural attributes impart excellent stability and durability, making such materials particularly suitable for applications requiring long‐term operational reliability, including catalysis and biosensing [115, 116, 117]. The high compositional uniformity and structural integrity further ensure reproducible physicochemical properties and consistent catalytic activity. Owing to the deterministic relationship between synthesis conditions and the resulting equilibrium structure, this strategy offers exceptional batch‐to‐batch consistency and process reproducibility. By precisely controlling interfacial energy and component distribution, thermodynamic driving forces can guide the formation of complex architectures [118]. When combined with theoretical modeling, this enables rational design of HEAzymes with tailored properties. As shown in Figure 3, this section systematically reviews key thermodynamic strategies, including high‐pressure, high‐temperature, and electrosynthesis methods to elucidate their roles in regulating the structure and catalytic performance of HEAzymes.

**FIGURE 3:**
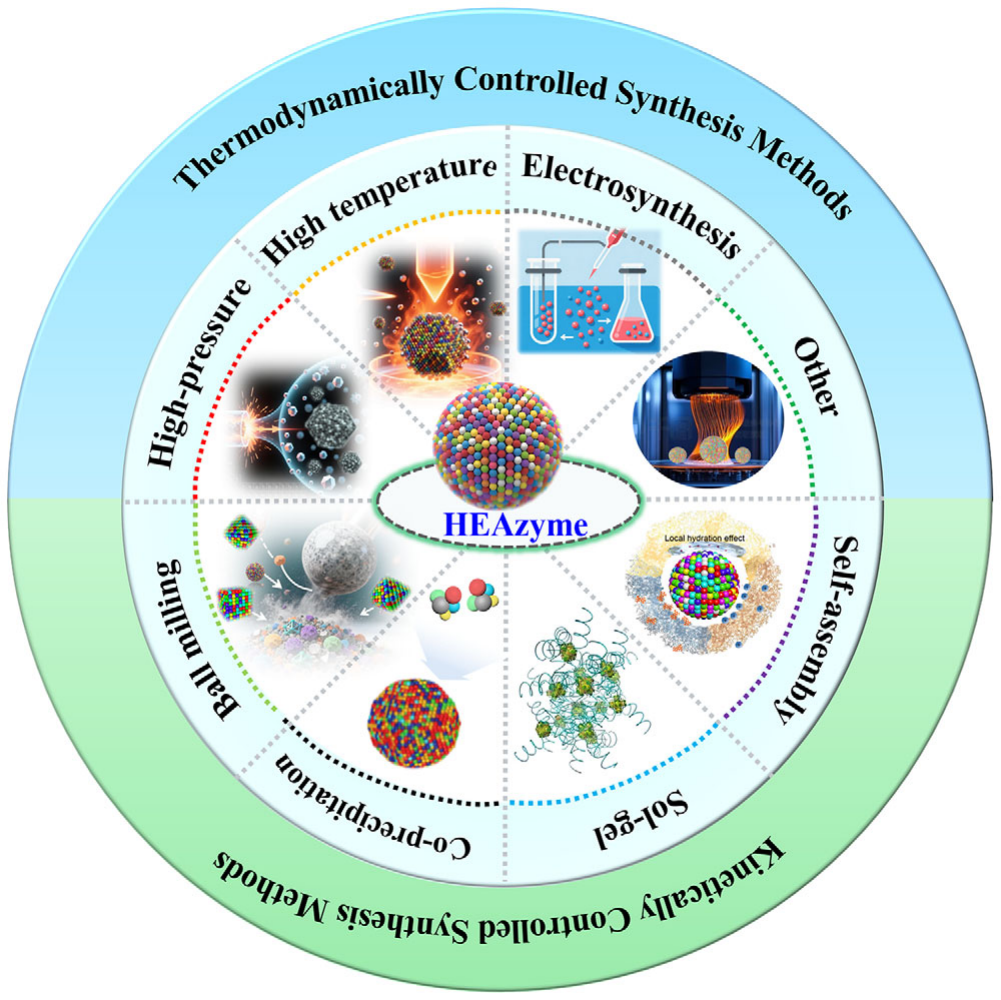
Thermodynamically and kinetically controlled synthesis routes for HEA NPs.

High‐temperature synthesis relies on elevated temperatures often near or above the materials melting point, to provide sufficient activation energy for atomic interdiffusion, overcoming miscibility gaps and achieving atomic‐scale mixing [119]. This process facilitates the growth of well‐crystallized solid solutions with equilibrium morphologies. The high‐temperature environment eliminates metastable phases and internal stresses, enhancing thermal stability and structural integrity [120, 121, 122]. While the method imposes stringent demands on high‐temperature equipment and may encounter challenges with stoichiometric deviations, it remains essential for producing HEAzymes with superior structural ordering and stability, particularly for catalytic applications under demanding conditions. A universal carbothermal shock strategy has been developed for ultrarapid alloying of up to eight distinct elements into single‐phase HEA NPs (Figure 4A) [71]. This approach subjects’ carbon‐supported metal salt precursors to controlled thermal pulses reaching ≈2000 K within ≈55 ms with heating rates of 10^5^ K s^−1^, followed by instantaneous quenching. The resulting molten droplets crystallize into homogeneous, aggregation‐free NPs with tunable composition, size, and phase through modulation of peak temperature and pulse duration. The methodology has demonstrated particular efficacy in circumventing predicted immiscibility barriers [123]. Recent this technology extensions include the fabrication of carbon‐supported HEO NPs [124], AuFeCoCuCr NPs [125], PtCoNiRuIr/C [126], and PtIrFeNiCoCe alloys [127]. Achieving uniform HEA NP dispersion on granular supports of carbon, alumina, zeolites represent a critical translational challenge. As show in Figure 4B, Skrabalak et al. present a versatile methodology for fabricating HEA NPs via a NPs conversion route [128]. This strategy entails the controlled colloidal preparation of biphasic core‐shell NPs, which undergo a thermal transformation at 800°C to yield single‐phase HEA NPs. The research delineates fundamental synthetic guidelines for precursor core‐shell structures. Upon achieving uniform core‐shell NPs, a facile annealing protocol that adaptable to diverse support materials was utilized to generate single‐phase HEA NPs incorporating Pd, Cu, Pt, Ni, Co, Au, Ag, and Sn. This investigation showcases tunable modulation of elemental interdiffusion between core and shell domains for synthesizing monodisperse HEA NPs. As show in Figure 4C, A fast‐moving bed pyrolysis strategy addresses this obstacle through wet impregnation combined with rapid pyrolysis at 923 K [129]. The instantaneous decomposition of metal chloride precursors generates numerous small nuclei by lowering nucleation free energy, effectively suppressing agglomeration. This scalable technique successfully anchors ultrasmall (≈2 nm), highly dispersed HEA NPs comprising up to ten immiscible elements of Mn, Co, Ni, Cu, Rh, Pd, Sn, Ir, Pt, Au onto diverse supports. Aerosol‐based strategies enable continuous, scalable HEA‐NP fabrication through droplet‐to‐particle conversion, offering distinct advantages in compositional complexity and structural control [130, 131, 132, 133]. As show in Figure 4D, spray pyrolysis with citric acid blowing agents generates hollow HEA The authors acknowledge theNPs via rapid thermal processing [134]. According to this method, continuous‐flow hydrocarbothermal synthesis produces sub‐2 nm carbon‐supported HEA NPs with loadings approaching 30 wt%, overcoming the conventional size‐loading trade‐off. Furthermore, Plasma jet processing achieves ultrafast in‐flight alloying at >5000 K with throughputs of ≈35 g h^−1^ [135]. Furthermore, liquid‐metal‐mediated biphasic dewetting enables self‐confined growth of HEA polyhedral with equilibrium morphologies, accelerating facet engineering efforts [136]. These methodologies collectively address scalability challenges while providing application‐tailored control: spray pyrolysis enables hollow architecture engineering and ultrafine particle synthesis; plasma processing delivers optical tunability and high throughput under extreme thermal conditions; and liquid‐mediated approaches provide morphological precision for facet engineering. Furthermore, additional high‐temperature strategies, including liquid shock techniques, in situ conversion methods that circumvent solubility product limitations, nonequilibrium approaches, and rapid Joule heating have been employed for HEA NPs fabrication [137]. Among these, carbothermal shock excels in ultrarapid alloying and overcoming immiscibility constraints. Strategic selection among these platforms should be guided by target application requirements: catalysis favors high surface‐area architectures or well‐defined active sites; energy conversion demands specific optical or electronic properties; and industrial implementation prioritizes reproducibility and manufacturing throughput.

**FIGURE 4:**
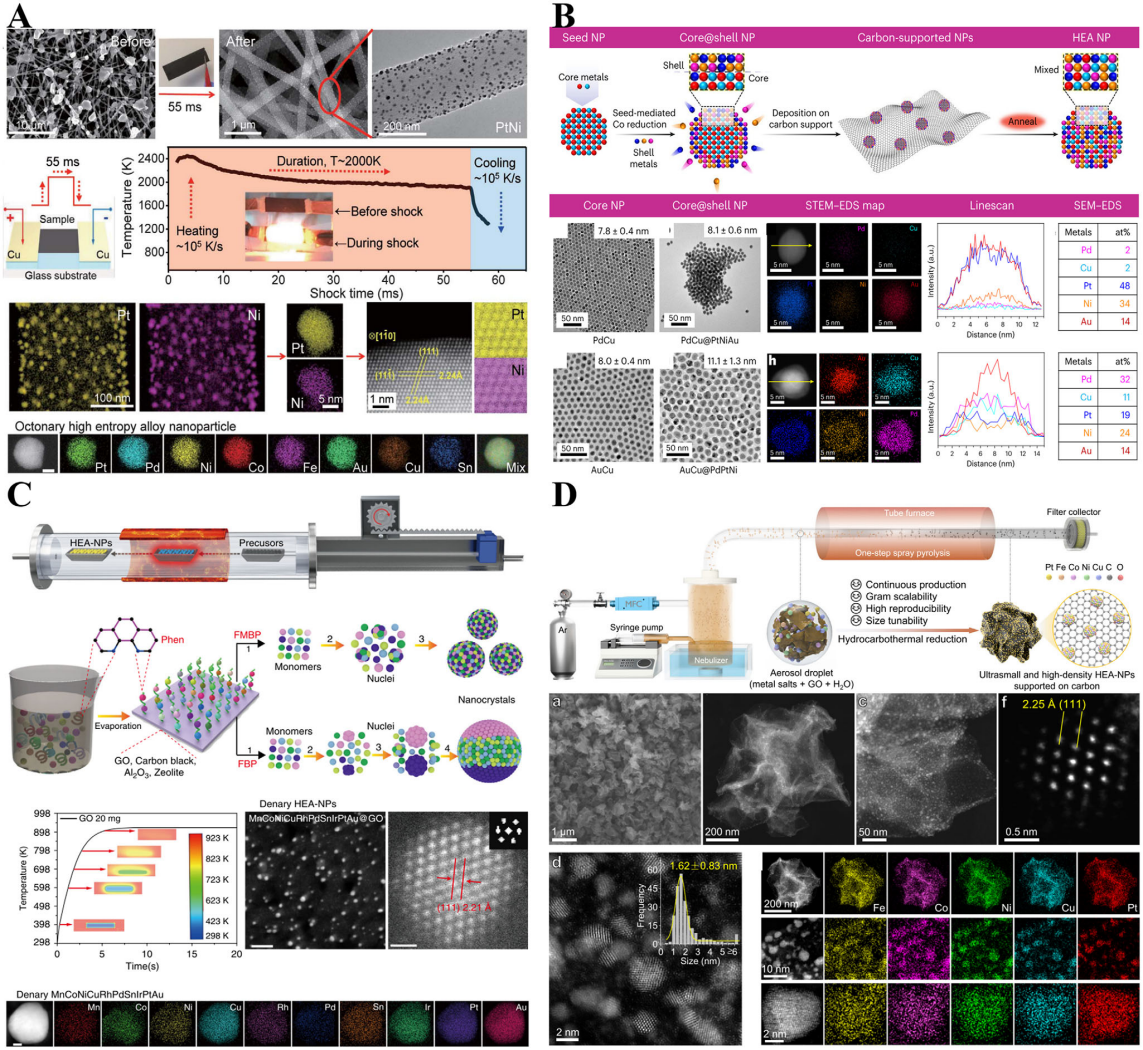
(A) Carbothermal shock synthesis of HEA NPs. Reprinted with permission from Ref. [71] Copyright 2018, Springer Nature. (B) Monodisperse HEA NPs were synthesized via a thermal annealing conversion pathway. Reprinted with permission from Ref. [128] Copyright 2024, Springer Nature. (C) Synthesis of HEA NPs on supports by fast‐moving bed pyrolysis. Reprinted with permission from Ref. [129] Copyright 2020, Springer Nature. (D) Hydrocarbothermal continuous‐flow spray pyrolysis synthesis of carbon‐supported HEA NPs. Reprinted with permission from Ref. [134] Copyright 2025, Springer Nature.

High‐pressure synthesis utilizes pressures ranging from several to tens of GPa to modulate the free‐energy landscape, driving phase transformations toward denser crystalline forms [138]. The applied pressure significantly reduces diffusion barriers, enabling the formation of homogeneous solid solutions from otherwise immiscible elements and stabilizing unconventional crystal structures. This method enhances solid solubility, promotes novel high‐entropy phase formation, and yields highly dense, crystalline products with minimal defects. It effectively suppresses elemental segregation and volatilization, ensuring compositional accuracy. Although the technique demands specialized pressure‐resistant equipment and typically produces limited sample sizes, its unique ability to access metastable high‐entropy structures makes it invaluable for exploring new nanozyme systems with distinctive catalytic properties suited for extreme environments. This approach was well‐illustrated in the realm of HEAs, where pressure can induce profound phase changes. For instance, Mao et al. demonstrated a pressure‐induced martensitic transformation in the canonical CrMnFeCoNi HEA, transitioning from a fcc to a hcp phase [139]. Initiated at approximately 14 GPa, this transformation is driven by the suppression of local magnetic moments, which destabilizes the original fcc lattice. Uniquely, this transition occurring over a broad pressure range up to 440 GPa, and the hcp phase is partially retained upon complete decompression. This results establishing high‐pressure treatment as a non‐conventional route for tailoring phase constitution and the functional properties of HEAs. Furthermore, high‐pressure strategies have proven pivotal in nanomaterial design, particularly in ultrafine HECs.

As shown in Figure 5A,B, Cao et al. have developed an economical synthetic route that integrates spray drying with thermal decomposition‐reduction, enabling the fabrication of 3D HEA nanoframeworks (HEA‐3DNFs) spanning quinary to octonary systems [140]. This approach accommodates noble metal‐based compositions, specifically those incorporating Pt, Ir, Ru, and their bimetallic PtRu variants. The simultaneous co‐reduction of multiple metallic precursors is achieved under gentle H_2_ heating, yielding homogeneous single‐phase solid solutions. Through in situ scanning transmission electron microscopy observations coupled with density functional theory (DFT) modeling, the researchers elucidated a structural transition from Pt‐rich face‐centered cubic to Ru‐dominant hexagonal close‐packed phases. Notably, the phase constitution can be deliberately tuned by modulating reduction temperatures and the relative Pt/Ru stoichiometry. These mechanistic insights provide valuable guidance for engineering noble metal HEA catalysts with enhanced performance and adjustable characteristics. He and colleagues developed a NaCl self‐assembly templating strategy where the application of high pressure during thermal treatment serves a dual purpose [141]. It suppresses atomic diffusion and phase segregation to ensure the formation of a homogeneous high‐entropy phase, while the template confines the material to prevent agglomeration. This approach enabled the universal synthesis a series of below 10 nm HECs, including nitrides, oxides, sulfides, and phosphates uniformly dispersed on porous carbon matrices. The versatility of this methodology, validated through advanced characterization and computational modeling, offers a promising avenue for the rational design and scalable fabrication of ultrafine HEMs.

**FIGURE 5:**
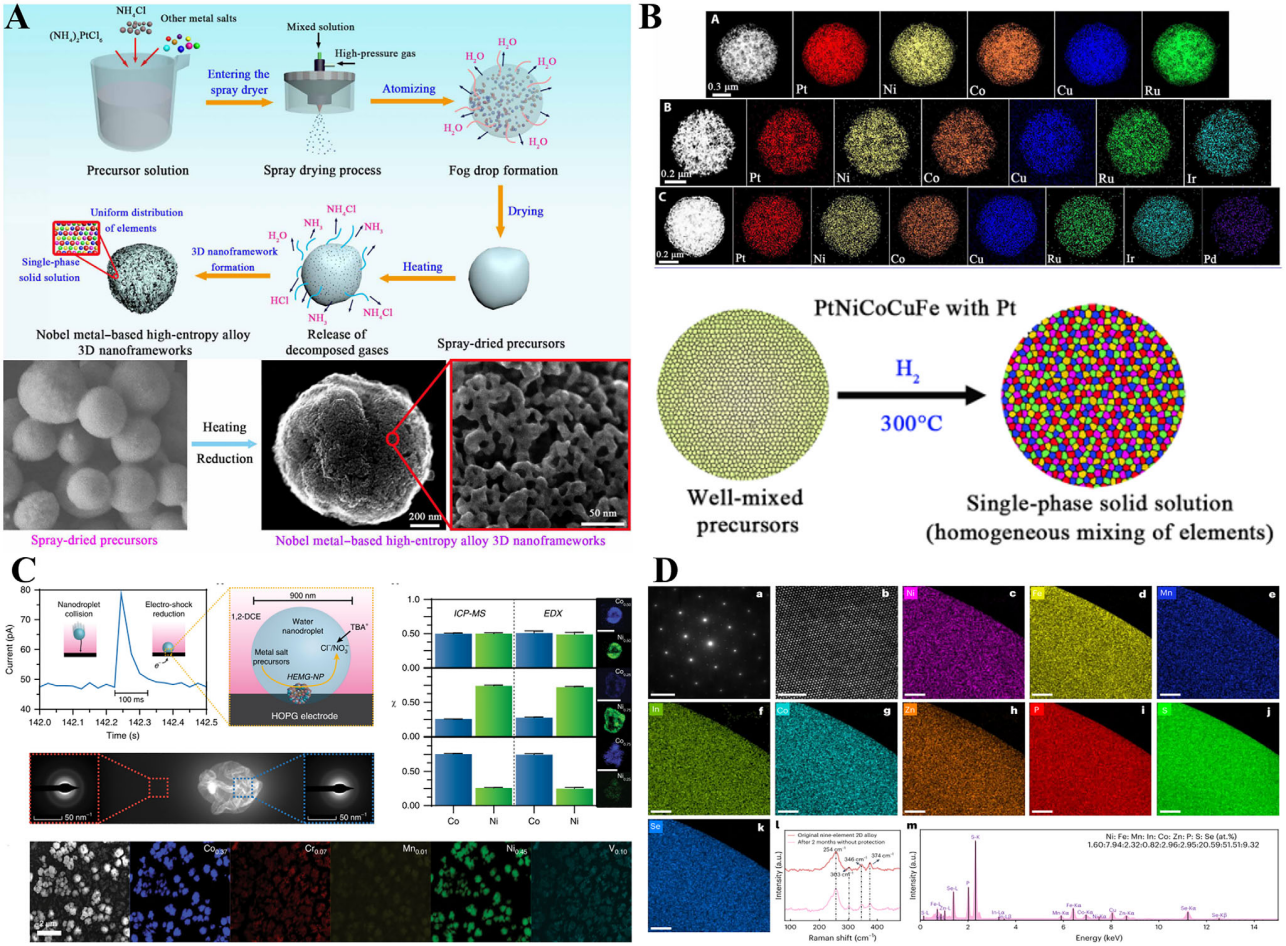
Thermodynamically controlled methods for synthesis of HEA NPs: (A‐B) Precise synthesis of targeted noble metal HEMs. Reprinted with permission from Ref [140]. Copyright 2025, AAAS. (C) Electrosynthesis of high‐entropy metallic glass NPs. Reprinted with permission from Ref [142]. Copyright 2025, Springer Nature. (D) Space‐confined chemical vapor transport strategy to synthesize 2D multicomponent materials. Reprinted with permission from Ref. [148] Copyright 2025, Springer Nature.

Electrosynthesis can controlled electrode potentials to drive the simultaneous deposition of multiple metallic elements, enabling the formation of HEAs or intermetallic compounds with atomic‐level homogeneity. Typically conducted in aqueous or ionic liquid systems at ambient temperatures, this approach offers low energy consumption and operational simplicity. The advantages include the capability to co‐deposit five or more elements, the formation of dense films or 3D architectures with tunable crystallinity, and the use of electrode potential as a fundamental thermodynamic parameter to steer the system toward equilibrium. Challenges primarily involve the need for matched deposition potentials, often addressed through ligand‐assisted coordination, as well as specific substrate requirements. Nevertheless, owing to its energy efficiency and superior compositional control, electrosynthesis has emerged as a promising strategy for fabricating HEAzymes, particularly in the fields of electrochemical biosensing and energy‐related applications. As shown in Figure 5C, Dick and colleagues developed an electrosynthesis route for the preparation of high‐entropy multimetallic glass NPs (HEMG NPs) [142]. This strategy confines up to eight equimolar metal salt precursors within water nanodroplets emulsified in dichloromethane. Upon collision of these nanodroplets with the working electrode, the constituent metals are simultaneously electrodeposited, yielding an amorphous microstructure characterized by intimately mixed atomic species. The stoichiometry of the metals can be precisely controlled by adjusting the precursor concentrations within the nanodroplets. Demonstrating their potential in energy conversion, CoFeLaNiPt HEMG‐NPs were applied to electrocatalytic water splitting and exhibited outstanding performance. In another approach, Zhang et al. employed a mechanical‐physical route in which metal microparticles were mixed and milled into HEA microflakes with a fcc crystal structure [143]. These microflakes were subsequently compressed into a target and processed in arc‐discharge equipment to synthesize HEA NPs. Using this method, a total of 16 types of HEA NPs that ranging from ternary to septenary compositions were successfully synthesized and characterized. Beyond metallic HEMs, the concept has also been extended to hybrid systems. For instance, a high‐entropy metal‐organic framework incorporating Fe, Ni, Co, Cu, and Y was synthesized via electrodeposition and subsequently employed as a template to fabricate a high‐entropy phosphide/carbon composite (FeCoNiCuYP/C) [144]. The resulting material features an amorphous structure rich in catalytically active sites. Combined theoretical calculations and experimental characterizations reveal that Co, Ni, and Fe play critical roles in modulating the electronic structure of the composite and optimizing the binding strength of reaction intermediates [145]. Collectively, this work presents an innovative route for designing efficient high‐entropy electrocatalysts with considerable promise for advanced electrocatalytic applications.

Beyond established synthetic routes, several emerging techniques have been developed to meet the growing demand for scalable, efficient, and structurally controlled fabrication of HEMs. These methods could distinct physical and chemical principles ranging from rapid thermal processing to laser‐induced synthesis [146] and confinement‐driven assembly [147]. The above offering complementary advantages in throughput, compositional versatility, and architectural precision. As shown in Figure 5D, Gong et al. introduce a spatially confined chemical vapor transport approach for fabricating high‐quality 2D mixed‐cation materials (2DMCMs) devoid of phase segregation and compositional gradients [148, 149]. Based on this technique, they successfully synthesize twelve distinct transition metal phosphorous chalcogenides and their alloyed derivatives, encompassing systems with up to nine constituent elements. The integration of chemical vapor transport with geometrically restricted growth environments enables homogeneous vapor‐phase distribution, precise process control, and preferential in‐plane crystal expansion. Such architectural precision facilitates exact stoichiometric regulation and elemental uniformity, even within HEA configurations. This synthetic paradigm for producing compositionally accurate, high‐fidelity 2DMCMs substantially expands the accessible material landscape for investigating structure‐property relationships and elucidating fundamental physicochemical phenomena in complex 2D systems. Microwave‐assisted synthesis exploits the rapid heating capabilities of carbonaceous substrates [150]. Using microwave‐absorbing supports such as reduced graphene oxide [151], carbon nanofibers [152], or carbonized wood [153], which temperatures exceeding 1400 K are achieved within seconds and enabling simultaneous precursor decomposition and molten‐state solidification. This strategy could yield uniform HEA NPs with controlled size and elemental distribution, and its compatibility with roll‐to‐roll processing makes it highly attractive for large‐scale manufacturing. Laser solid‐phase synthesis utilizes laser irradiation to induce localized heating on a laser‐induced graphene support [154]. The process involves a cascade of events ultimately producing graphene‐encapsulated NPs. This method is remarkably versatile, extending to HEOs, sulfides, and phosphides, with production rates reaching up to 30 g h^−1^ under optimized conditions. Self‐lattice framework‐guided synthesis enables phase‐selective fabrication of ultrathin HEOs [155]. By incorporating gallium as an assistor to lower formation energy, this method allows controlled synthesis of desired phase structures, including rock‐salt, spinel, perovskite, and fluorite phases. The resulting ultrathin spinel oxides exhibit excellent catalytic activity and stability in oxygen evolution reactions, underscoring the importance of phase engineering in optimizing functional properties [156]. Liquid metal nanoreactor strategy offers a conceptually distinct approach for constructing HEA arrays with precise spatial organization [157]. Capitalizing on the natural tendency of liquid metals to minimize surface energy, this method creates a self‐confined environment that directs nucleation and growth to predetermined locations. The low diffusion energy barrier and high diffusivity inherent to liquid metal systems facilitate efficient aggregation, enabling uniform HEA arrays comprising up to eleven constituent elements. These arrays demonstrate promising holographic responses across a broad spectrum, highlighting their potential in optical and sensing technologies. These emerging strategies significantly expand the methodological landscape for HEMs. Each approach brings unique strengths, whether in scalability, throughput, phase control, or spatial patterning underscoring the growing maturity and diversification of the field. Their continued development is expected to accelerate the discovery and application of HEMs across catalysis, energy conversion, optics and beyond.

It is worth noting that the synthesis of conventional HEMs is typically optimized for bulk phase stability, mechanical properties, or macroscopic catalytic activity, relying primarily on thermodynamic driving forces to form high‐entropy solid solutions or on kinetic trapping to stabilize meta‐stable multicomponent structures [158]. However, extending these general principles directly to HEAzyme systems necessitates systematic adjustments to accommodate the distinctive interface chemistry and biological application requirements inherent to nanozymes. Specifically, the catalytic activity of HEAzymes is critically governed by the coordination environment of surface atoms, defect density, and exposed crystal facets. Consequently, synthetic strategies must incorporate surface engineering approaches, such as selective etching, ligand modulation, or post‐synthetic annealing to directionally tailor the local coordination configurations and electronic structures of surface elements, rather than merely maximizing bulk configurational entropy [159]. Moreover, HEAzymes intended for biological applications must maintain long‐term dispersion stability in physiological buffers or cell culture media [160]. This demands the incorporation of biocompatible stabilizers or the construction of surface charge layers to provide steric hindrance and electrostatic repulsion. Accordingly, mild liquid‐phase co‐reduction and microemulsion techniques should be adopted in lieu of conventional high‐temperature solid‐state or carbothermal reduction methods to ensure colloidal stability. In addition, whereas traditional HEM synthesis often employs high temperatures, organic solvents, or toxic precursors, HEAzymes must satisfy stringent biosafety standards. Synthetic routes should therefore prioritize green reducing agents or biomineralization strategies to eliminate residual heavy metal ions and minimize the use of strong reducing agents, while preserving surface functional groups amenable to subsequent bioconjugation with antibodies, peptides or other biomolecules. Most importantly, the enzyme‐mimetic behavior of HEAzymes is intimately linked to NPs’ size, morphology, and porosity. The synthetic paradigm must therefore shift from a conventional phase‐formation‐oriented approach to one guided by active‐site accessibility and mass‐transfer efficiency, leveraging tempting, Ostwald ripening regulation, or self‐assembly to achieve precise morphological and dimensional control [161]. Through these multidimensional adjustments, the principles of entropy engineering and kinetic trapping inherent to conventional HEM synthesis are effectively reoriented at the nanoscale, bridging the gap between macroscopic materials fabrication and nanozyme biological function, and thereby establishing clear synthetic guidelines for design of HEAzymes.

### 2.2 Kinetically Controlled Synthesis Methods

In contrast to thermodynamic control which pursues stability and equilibrium, kinetic control in HEAzymes synthesis aims to capture and stabilize metastable, non‐equilibrium nanostructures by precisely regulating the rate and pathway of the reaction process. The essence of this strategy lies in utilizing methods such as rapid precursor injection, low‐temperature environments, or selectively adsorbing ligands to deliberately prevent the system from reaching thermodynamic equilibrium, thereby freezing the reaction at a higher‐energy intermediate state and obtaining materials [162, 163, 164]. The primary advantage of this approach lies in the precise and versatile control over morphology and size, together with the construction of heterostructures that are inaccessible under equilibrium conditions. Moreover, it generates highly active sites abundant in defects and characterized by high‐index facets and lattice strain, thereby markedly enhancing catalytic performance and enabling fine‐tuning of material properties. Additionally, it broadens the accessible material space and facilitates rapid, energy‐efficient synthesis routes [165, 166, 167]. Kinetic control synthesis serves as a powerful tool for function‐oriented material design, allowing researchers to break through thermodynamic limitations and create nanomaterials with new structures and properties, greatly advancing the field of HEAzymes. Based on this, this section will systematically review recent advances in the study of HEAzymes based on kinetic control strategies, including ball milling, co‐precipitation, sol‐gel, self‐assembly and biosynthetic methods.

Ball milling utilizes mechanical energy to drive physicochemical reactions between solid precursors, combining top‐down fragmentation with bottom‐up alloying. Typically conducted under solvent‐free or minimal‐solvent conditions without the need for extreme temperatures or pressures, this method demonstrates broad applicability to refractory metals and ceramics. As a representative kinetically controlled process, ball milling facilitates the formation of metastable high‐entropy structures, including amorphous phases and supersaturated solid solutions. By mechanically inhibiting atomic diffusion while introducing lattice defects. Owing to its scalability and exceptional capability, ball milling remains indispensable for synthesizing HECs. Researchers use ball milling to synthesize HEMs with tailored functionalities [168]. The high‑entropy matrix provides structural stability under humidity. For metallic systems, high‑energy ball milling produces Nb‑Cr‑Ta‑V‑W refractory HEA NPs via top‑down fragmentation, leading to a size‑dependent transition from bulk diamagnetism to superparamagnetic, offering low‑cost magnetic materials for harsh environments [169]. Beyond single‑stage milling, multistage strategies on FeCoNiCr_0.4_Cu_0.2_ HEA powders yield high‑aspect‑ratio particles with thin amorphous intergranular layers, achieving temperature‑stable permeability, broadband electromagnetic wave absorption, and corrosion resistance [170]. Ball milling also extends to functional coatings: doping Zn/Cu into a quinary spinel‑type HEO produces hexanary compositions with enhanced infrared emissivity; when incorporated into ceramic glazes, they deliver high infrared radiation and dual antibacterial activity [171]. As shown in Figure 6A, in a cubic Zr_0.5_(NiFeCuMnCo)_0.5_O_x_ host, reversible exsolution and dissolution of metallic species were observed under redox cycling, driven by a dominant entropic contribution to the Gibbs free energy [172]. This self‐regeneration behavior confers exceptional thermal stability during long‐term CO_2_ hydrogenation, highlighting the potential of entropy‐driven processes facilitated by the nonequilibrium structures achievable via ball milling for designing sinter‐resistant catalytic materials. Collectively, these studies underscore the versatility of ball milling and related mechanical processing strategies in synthesizing HEMs with tailored structures and properties. The ability to control microstructural features such as crystallite size, defect concentration, and phase stability, combined with the intrinsic advantages of HECs, positions these methods as powerful tools for advancing functional materials across many applications.

**FIGURE 6:**
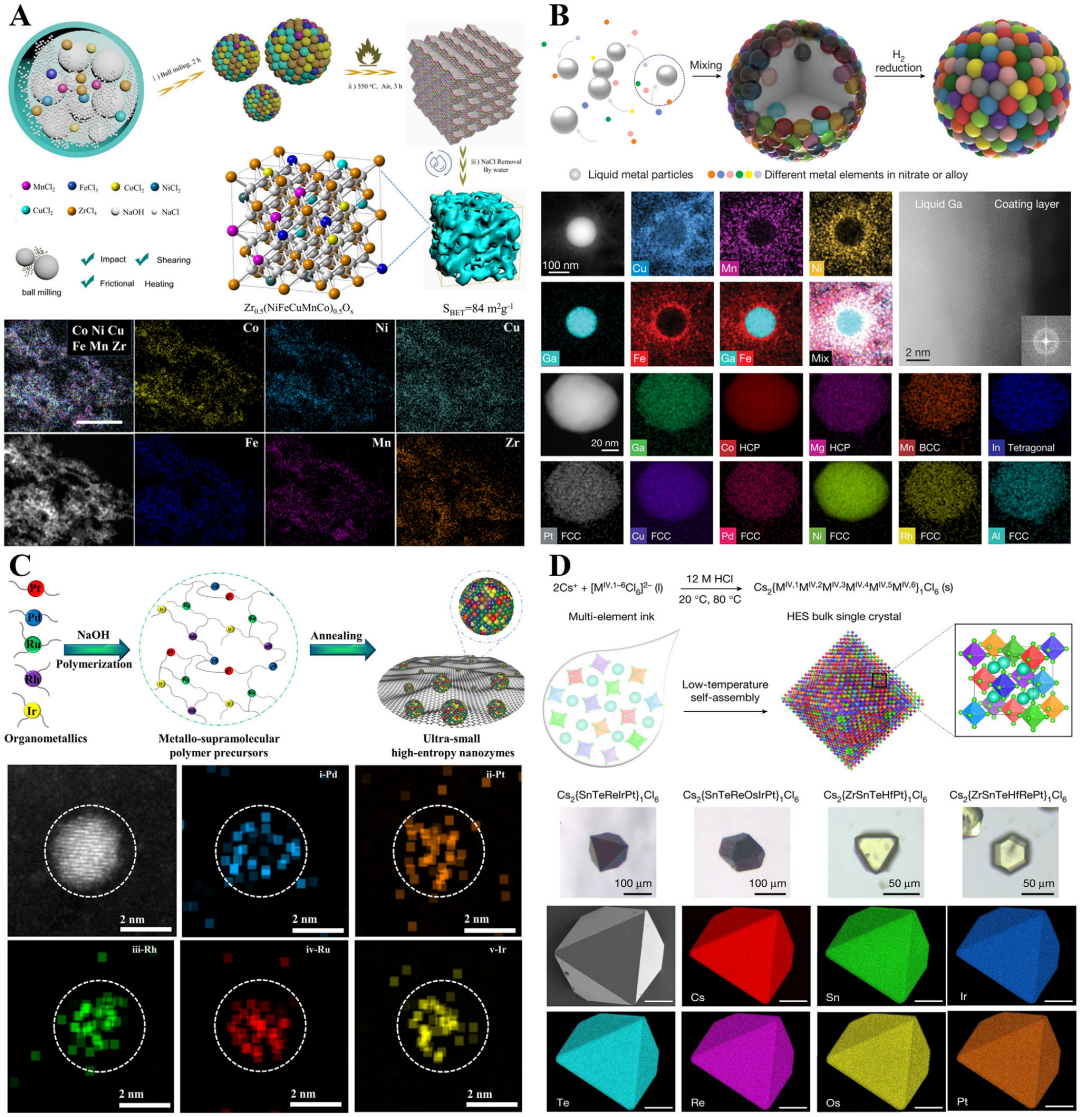
Kinetically controlled methods for synthesis routes for HEA NPs: (A) Ball milling fabricated transition metals‐based HEA NPs via self‐regeneration process. Reprinted with permission from Ref [172]. Copyright 2021, Wiley‐VCH. (B) Liquid metal for synthesis of HEA NPs. Reprinted with permission from Ref. [184] Copyright 2025, Springer Nature. (C) Metal‐ligand cross‐linking strategy for fabrication of HEA NPs. Reprinted with permission from Ref. [45] Copyright 2023, Wiley‐VCH. (D) Room‐ and low‐temperature driven self‐assembly of multielement inks to high‐entropy semiconductor. Reprinted with permission from Ref [212]. Copyright 2023, Springer Nature.

Solution‐based synthesis approaches achieve atomic‐scale mixing of multiple metal ions through concurrent nucleation and precipitation. By regulating parameters such as precursor concentration, pH, and titration rate, these methods ensure high compositional uniformity and promote the formation of single‐phase high‐entropy solid solutions [173]. The rapid precipitation kinetics effectively freeze hetero‐elements within nanocrystalline lattices, overcoming thermodynamic miscibility gaps. Although scalable and operable under ambient conditions, maintaining uniform precipitation kinetics across all components remains challenging; the use of complexing agents or optimized mixing protocols is often necessary to mitigate elemental segregation. Subsequent thermal treatment typically converts the precursors into well‐crystallized HEOs or sulfides. Building upon these core principles, particularly the emphasis on atomic‐level mixing, kinetic control, and compositional uniformity, a variety of solution‐based strategies have been developed to produce HEA and HEO nanomaterials with tailored size, morphology, and composition [174, 175, 176, 177]. These strategies can be broadly categorized into oil‐phase reduction, solvothermal synthesis, hydrothermal routes, and postsynthetic transformation approaches.

Oil‐phase reduction methods exploit the immiscibility between aqueous and organic phases to achieve controlled nucleation and growth. In a representative approach, PdPtRuRhAu HEAs were synthesized through a three‐stage process: homogeneous mixing of metal salts via capillary reinforcement, rapid solvent vaporization upon injection into a high‐temperature oil phase, and cooperative reduction. By systematically varying these parameters, a series of size‐tunable HEA NPs (2.81–5.74 nm) was obtained. In a related study, Wang et al. demonstrated the simplicity of this approach by preparing uniform Pt_18_Ni_26_Fe_15_Co_14_Cu_27_ NPs via a one‐pot oil‐phase reaction at 220°C, yielding particles of approximately 3.4 nm [178]. These examples illustrate how the solution‐based principle of concurrent nucleation can be extended to multicomponent systems under mild, scalable conditions.

Solvothermal synthesis offers enhanced control over reaction kinetics and phase formation using pressurized, elevated‐temperature solvents. Iversen and colleagues developed a versatile solvothermal protocol that yields phase‐pure bimetallic and HEAs across the entire compositional space [179]. By tailoring the solvent system and precursor chemistry, they synthesized six fully miscible bimetallic systems, including Pd_x_Ru_1‐x_, Pt_x_Ru_1‐x_, and Ir_x_Ru_1‐x_ all adopting a fcc structure, a phase previously unreported for ruthenium‐rich systems. Extending this approach to PtIrPdRhRu HEAs, they discovered that the mixed system reduces uniformly at about 200°C [180]. This synergy is attributed to autocatalytic reduction on crystal planes during nucleation, which synchronizes the incorporation of all five elements into a single‐phase alloy. The same solvothermal principles have been applied to oxide systems. Wang's group reported the low‐temperature synthesis of high‐entropy (Co, Cu, Fe, Mn, Ni)_3_O_4_ spinel oxides via solvothermal synthesis followed by annealing [181]. This work represents the successful low‐temperature fabrication of HEOs, yielding NPs of approximately 5 nm. In parallel, wet‐chemical approaches have enabled morphological control beyond simple spherical NPs [182]. Using an oleylamine‐based system with cetyltrimethylammonium chloride as a structure‐directing agent, PtIrRhCoFeNiCu HEA nanodendrites were synthesized with nearly 100% selectivity for the dendritic morphology [74]. The resulting nanocrystals feature high‐index crystal facets, illustrating how solution‐phase synthesis can simultaneously achieve compositional complexity and architectural sophistication. Hydrothermal synthesis provides an alternative route that operates under milder conditions while exploiting in situ chemical transformations. Gao et al. developed a hydrothermal method under 170°C that yields compositionally uniform HEA NPs by leveraging reactive hydrogen radicals generated from organic dehydrogenation on pre‐formed nuclei [183]. These radicals create transient non‐equilibrium microenvironments within an otherwise near‐equilibrium solution, relaxing thermodynamic and kinetic constraints to enable simultaneous reduction of all metal precursors. This demonstrating how solution‐based kinetic control can be harnessed to optimize functional properties. As shown in Figure 6B, Fu et al. demonstrate that liquid metals exhibiting negative enthalpy of mixing with disparate metallic species can establish thermodynamically favorable environments while serving as effective dynamic reservoirs for atomic interdiffusion [184]. This enables the fabrication of HEA‐NPs incorporating markedly heterogeneous elements under moderate synthetic conditions. The constituent elements span extensive ranges of atomic dimensions and thermal transition temperatures. Furthermore, structural precision at the nanoscale is achieved through systematic modulation of mixing enthalpy parameters. In situ characterization techniques successfully capture the temporal evolution from liquid metallic precursors to crystalline HEA‐NPs, revealing dynamic fission‐fusion phenomena throughout the alloying trajectory. Beyond direct synthesis, post‐synthetic modification represents a complementary solution‐based strategy for expanding structural diversity [185, 186]. Schaak and colleagues developed a route to convert colloidal HEA NPs, such as NiPdPtRhIr, NiFeCoPdPt, and NiFePdPtIr into high‐entropy intermetallic, including Zn‐based γ‐brass, In‐based CsCl, and Sn‐based NiAs‐type structures [187]. This approach exploits the inherent atomic homogeneity of HEA precursors to access ordered intermetallic phases that are otherwise difficult to synthesize directly, while preserving multifaceted morphology, underscoring the versatility of solution‐based platforms for post‐synthetic structural evolution. Collectively, oil‐phase reduction, solvothermal synthesis, hydrothermal routes, and post‐synthetic transformation demonstrate the remarkable adaptability of co‐precipitation and related wet‐chemical strategies. Grounded in atomic‐level mixing, kinetic control, and compositional uniformity, these methods enable precise tuning of composition, size, morphology, and crystal structure under mild conditions, offering robust platforms for engineering HEMs across diverse applications spanning catalysis, energy conversion and the like [188, 189, 190, 191].

The sol‐gel process enables molecular‐level homogenization of multicomponent precursors through hydrolysis and condensation reactions, forming a 3D gel network that immobilizes elements at the atomic scale. This method provides precise stoichiometric control and is particularly well suited for synthesizing HEOs with high specific surface areas and porous structures [192, 193]. However, divergent hydrolysis rates of metal alkoxides can lead to elemental heterogeneity, necessitating kinetic modulation via pH adjustment, chelation, or non‐hydrolytic routes. Owing to its low‐temperature operation and excellent compositional control, the sol‐gel method has become a versatile platform for fabricating uniform HEMs. Building upon these core attributes, namely molecular‐level mixing, precise stoichiometry, and tunable reaction kinetics the sol‐gel method and its derived strategies have been extensively employed to access HEMs with tailored compositions, structures, and functionalities. The following examples illustrate the versatility of this method across oxide synthesis, catalytic applications, and compositional design. The inherent ability of the sol‐gel method to achieve atomic‐scale homogeneity makes it particularly effective for synthesizing HEOs with complex crystal structures. Wang et al. employed a polyacrylamide‐gel route, a variant of the sol‐gel approach to prepare high‐entropy spinel oxides in which five equimolar transition metals collectively occupy the Ni sites of NiAl_2_O_4_, yielding (Ni_0.2_Zn_0.2_Mg_0.2_Cu_0.2_Co_0.2_)Al_2_O_4_ after calcination at 800°C [194]. Systematic characterization confirmed phase purity, mixed valence states, and a porous morphology. This work exemplifies how the sol‐gel method enables precise control over cation distribution in complex oxide lattices. Beyond oxide synthesis, the compositional flexibility inherent to the sol‐gel method has been applied to engineer HEMs with optimized catalytic properties through rational compositional design. Ma et al. reported a room‐temperature, acid‐catalyzed sol‐gel route to prepare homogeneous high‐entropy electrocatalysts for methanol and urea electro‐oxidation [195]. Taking advantage of the sol‐gel method's precise stoichiometric control, they systematically tuned the ratios of Ni, Co, and Fe to optimize catalytic performance. In this system, nickel served as the primary active component, while cobalt reduced overpotentials and iron enhanced catalytic activity and durability. This work demonstrates how the compositional flexibility and molecular‐level mixing afforded by the sol‐gel method can be harnessed to design multifunctional high‐entropy electrocatalysts for energy conversion and value‐added chemical production. These studies underscore the versatility of the sol‐gel method as a foundational platform for synthesizing HEMs with precise compositional control and tailored functionality. By leveraging its inherent advantages of molecular‐level homogeneity, low‐temperature processing, tunable kinetics, and precise stoichiometric control, the sol‐gel method provides a powerful and adaptable route for developing advanced HEOs and electrocatalysts across a broad range of applications, from environmental remediation to energy conversion.

Self‐assembly exploits programmable interparticle interactions, such as van der Waals forces, hydrogen bonding, and electrostatic interactions to organize building units into long‐range ordered superlattices or hierarchical architectures [196]. Although thermodynamically driven, the process remains kinetically tunable, yielding materials with well‐defined symmetry, adjustable lattice parameters, and sharp interfaces. The primary challenge involves matching the size, shape, and surface chemistry of diverse nanocomponents to prevent random aggregation; surface ligand engineering is frequently employed to balance interaction forces and guide co‐assembly [197]. This method offers a distinctive route for creating HEMs with collective properties applicable to photonic, catalytic, and biomedical technologies. Building upon these core principles, particularly programmable interparticle interactions, kinetic tunability, and surface engineering, a variety of self‐assembly strategies have been developed to construct HEMs with controlled architectures, compositions and functionalities [198, 199, 200]. The following examples illustrate the versatility of this approach across interfacial assembly, soft‐templating, metal‐ligand cross‐linking, and surface engineering. Interfacial self‐assembly enables the formation of ultrathin high‐entropy structures with atomic‐scale uniformity. Fan et al. developed a naked‐metal‐ion self‐assembly protocol that converts bare aqueous ions directly into 1‑nm thick RuIrFeCoCrO_2_ HEO nanosheets at the salt interface [201]. In this process, molten salts strip hydration shells to generate desolated species that migrate freely; subsequent water evaporation drives them to the air‐liquid boundary for instant oxidation and crystallization. In a complementary approach, cathodic plasma electrolysis deposition was employed to fabricate FeCoNiMnCu HEA dendrites self‐assembled from NPs smaller than 40 nm [202]. The resulting 3D porous architecture, combined with highly defective lattices. Collectively, these examples illustrate how interfacial and plasma‐assisted self‐assembly can produce hierarchically structured HEMs for energy applications. Soft‐templating strategies utilized self‐assembled block copolymers as structure‐directing agents to create mesoporous HEMs with enhanced mass transport properties. Yamauchi et al. designed a one pot wet chemical reduction approach to synthesize core–shell PtPdRhRuCu mesoporous nanospheres using a diblock copolymer template [203]. The resulting nanospheres feature adjustable compositions and exposed porous architectures rich in HEA sites. They exhibit exceptional hydrogen evolution reaction activity in alkaline, acidic, and neutral electrolytes. This performance is attributed to synergistic interactions among multiple metal sites combined with efficient mass and electron transport enabled by the mesoporous structure.

Metal‐ligand cross‐linking has emerged as a versatile platform for constructing hierarchical high‐entropy and medium‐entropy nanostructures with diverse morphologies and functionalities [204, 205, 206]. Using ferric ion‐dopamine coordination, we have fabricated hierarchical superstructures with complex morphologies, including flower‐like, octopus‐like, and hedgehog‐like architectures through a multistep pathway involving pre‐cross‐linking, aggregation, sintering, polymerization, and final self‐assembly [207, 208]. This platform was extended to synthesize PtPdRuRhIr ultra‐small HEA NPs via aldol condensation of organometallics (Figure 6C) [45]. These NPs function as bifunctional nanoplatforms, combining excellent POD‐like activity with high photothermal conversion efficiency under near‐infrared (NIR) irradiation. Furthermore, surface engineering provides an additional lever for optimizing the catalytic performance of self‐assembled HEMs. PtRuFeCoNi HEA nanowires were surface‐engineered with polydopamine to form HEA NWs@PDA nanozymes with superior catalytic and photothermal properties [209]. DFT calculations revealed that polydopamine surface engineering moderately lowers the d‐band center, optimizes surface charge distribution, and enhances substrate adsorption‐desorption efficiency. These nanozymes were synergistically integrated with hydrogels for biosensing applications, demonstrating the potential of surface‐engineered self‐assembled HEMs in catalytic sensing. At the sub‐nanometer scale, HEAzymes system capable of endogenous targeted catalysis and enhanced tumor photothermal therapy [210, 211]. RuRhPtIrMo sub‐nanometer HEAzymes with approximately 8–10 atoms in thickness were first prepared via reduction‐diffusion and grafting methods. This nanozyme exhibits excellent POD‐like activity and high NIR absorbance, enabling efficient photothermal conversion and ROS generation under NIR irradiation. This work offering a perspective on sub‐nanometer HEAzymes fabrication and biomedical applications.

The self‐assembly concept has also been extended to high‐entropy semiconductors. Yang et al. developed solution‐based synthesis procedures for metal halide perovskite high‐entropy semiconductor single crystals based on the cubic Cs_2_MCl_6_ vacancy‐ordered double‐perovskite structure (Figure 6D) [212]. The synthesis relies on the self‐assembly of stabilized complexes in multielement inks, where free Cs^+^ cations and five or six different isolated MCl_6_^2−^ octahedral molecules are well‐mixed in strong hydrochloric acid. The resulting single‐phase crystals maintain the Cs_2_MCl_6_ structure with multiple elements randomly occupying the M‐site, producing complex vibrational and electronic structures with energy transfer interactions between confined exciton states. This work demonstrates the applicability of self‐assembly principles to high‐ entropy semiconductors, opening new avenues for optoelectronic and energy‐related applications. These studies underscore the remarkable versatility of self‐assembly as a foundational platform for constructing HEMs with precisely controlled architectures across multiple length scales. Based on its inherent advantages of programmable interparticle interactions, kinetic tunability, surface ligand engineering, and compatibility with diverse synthesis conditions, self‐assembly provides a powerful and adaptable route [213].

Recently, emerging synthetic strategies have been developed to address key challenges in the fabrication of HEA NPs, particularly regarding sustainability, ambient‐condition operation, and compositional control [214]. Among these, biosynthesis has gained attention as a sustainable approach that leverages microorganisms, plant extracts, or biomacromolecules to reduce and stabilize metal ions under benign aqueous conditions. In this process, biomolecules function not only as reducing agents but also as templates and morphology‐directing agents, facilitating the formation of structurally unique high‐entropy nanostructures. Despite its advantages in energy efficiency and environmental compatibility, this method faces limitations in controlling crystallinity, size distribution, and stoichiometry, with potential interference from residual biotemplates further constraining reproducibility and performance optimization. Nonetheless, biosynthesis remains a promising sustainable route for producing HEAzymes in biomedicine and environmental remediation, where biocompatibility and sustainability are of primary importance. In a distinct approach, Peng and coworkers employed hydrogen cold plasma to synthesize a series of Pt‐based binary and PtCrTaVFeAl HEA NPs under ambient conditions [215]. The method led to hydrogen anions generated in the plasma, which possess an exceptionally strong reducing ability, enabling the simultaneous reduction of Pt and reactive metal precursors. The resulting NPs were confirmed to form alloys and exhibited enhanced activity and durability in the methanol oxidation reaction compared to commercial Pt catalysts. This work provides an effective and universal route for synthesizing Pt‐containing binary and HEA NPs, establishing a new platform for the investigation of such nanomaterials.

For HEAzymes, the synthesis‐induced structural features of crystal phase, surface defect density, elemental distribution, particle size, and specific surface area are related to their enzyme‐like activities [216, 217, 218]. The crystal phase dictates the thermodynamic feasibility and selectivity of catalytic pathways by governing surface atomic arrangements, local coordination environments, and electronic band structures [219]. It is reported that the fcc phases facilitate electron transfer, thereby enhancing SOD‐like activity, whereas open‐framework perovskite phases promote O_2_ desorption, favoring CAT‐like reactions. Surface defects, particularly oxygen vacancies, serve as under‐coordinated active sites essential for H_2_O_2_ activation and O_2_^•−^ adsorption and disproportionation; however, defect densities must be carefully moderated to prevent structural degradation and loss of selectivity. The uniformity of elemental distribution directly governs the extent of multielement synergy: a homogeneous solid solution enables continuous modulation of electronic structures and adsorption energies, whereas surface segregation can deactivate active sites or compromise selectivity. Reducing particle size to the nanoscale significantly increases the proportion of under‐coordinated surface atoms and typically lowers the *K*_m_, indicating enhanced substrate affinity; nevertheless, ultrafine particles may suffer from diminished substrate binding due to pronounced quantum confinement effects or surface energy driven aggregation. Specific surface area influences apparent activity by determining active‐site accessibility and mass‐transfer efficiency; hierarchically porous architectures are generally preferable to a merely high surface area, as they better balance substrate accessibility with diffusional transport [220]. These structural parameters are not independent but mutually coupled. Consequently, the rational design of HEAzymes necessitates synergistic thermodynamic and kinetic control to holistically optimize these interdependent factors, rather than maximizing any single parameter, thereby enabling precise regulation of POD‐, SOD‐, and CAT‐like activities.

## 3 Determination of HEAzymes

Establishing whether the synthesized nanomaterial qualifies as HEAzyme is foundational to research in this field. As shown in Figure 7, the high entropy effect means that compared to pure metals and intermetallic compounds, the HEAs mixed conformational entropy enhanced. As the number of components increases, the mixed conformational entropy will also increase [221, 222]. The increased mixed conformational entropy facilitates the formation of stable single‐phase solids rather than friable intermetallic compounds and leads to excellent stability. The lattice distortion effect, which arises from the large difference in sizes of atoms to form the HEAs [223]. The random occupation of metal atoms in the lattice leads to severe lattice distortions. This severe lattice distortion not only significantly increases the hardness of the HEAs, but also decreases the electrical and thermal conductivity [224]. Slow diffusion effect shown that the slow diffusion effect is related to the lattice distortion effect [225]. Severe lattice distortion in HEAs increases the energy barrier for atomic diffusion. This effect also contributes to the formation of nanoscale HEAs due to the hindrance of surface atomic diffusion [119]. The cocktail effect implies a synergistic effect between the various elemental components of the HEAs, which results in a variety of unexpected properties [226]. The synergy endows them with vast compositional spaces and complex surfaces that provide abundant active sites and continuously tunable adsorption energies. The identification of HEAzymes relies on the systematic verification of three interconnected aspects: composition, structure, and elemental distribution.

**FIGURE 7:**
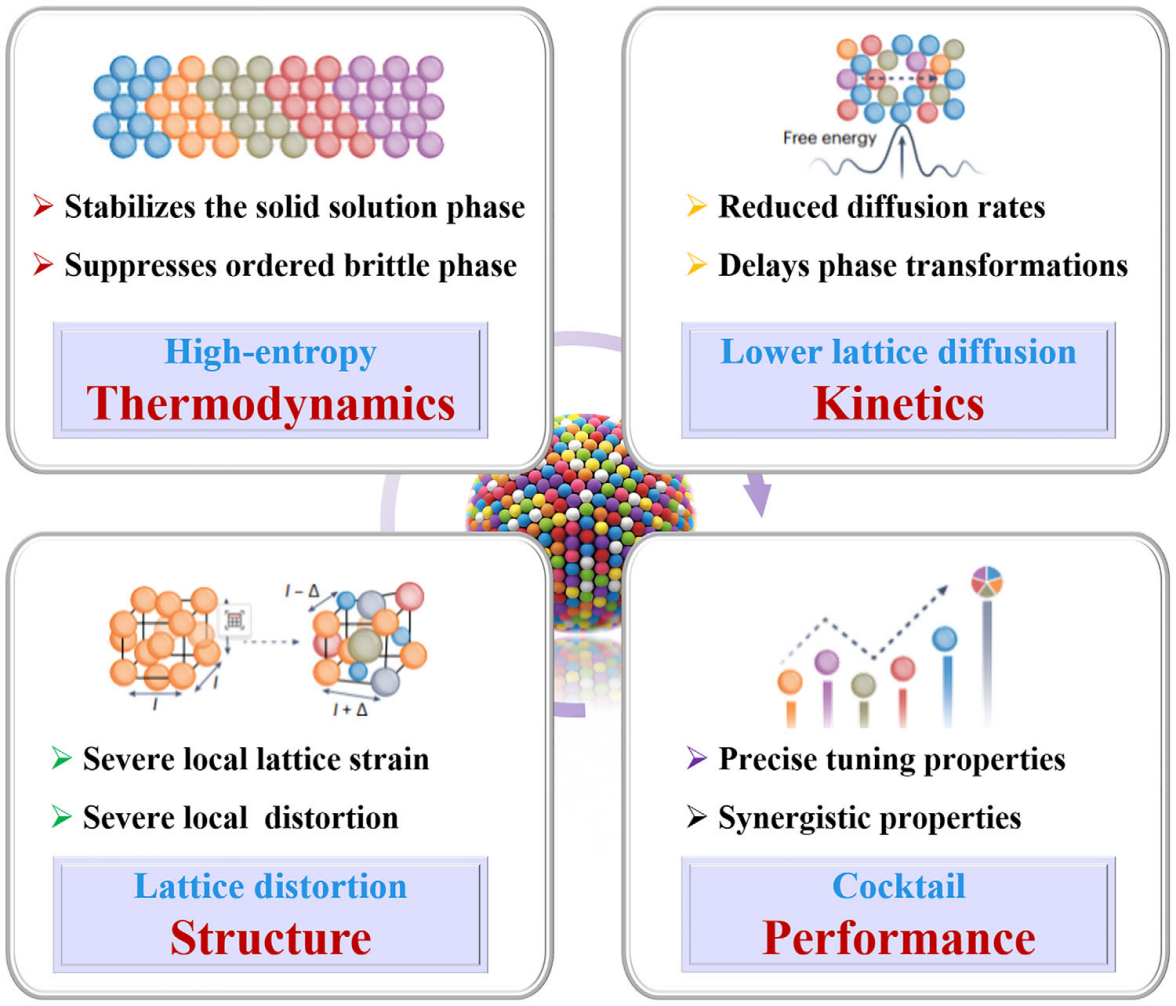
The unique high‐entropy, sluggish diffusion, lattice distortion and cocktail effects of HEAzyme.

As shown in Figure 8A,B, the composition must satisfy the core definition of a high entropy system, typically comprising five or more principal metal elements in near‐equal atomic ratios, with each element's atomic percentage ranging between 5% and 35%. The nearly unlimited combinations of elements make it extremely difficult to predict, evaluate, and assess the synthesizability of target system. The phase stability of multicomponent systems is generally evaluated through the Gibbs free energy of mixing (Figure 8C,D):

$$\Delta G_{\mathrm{mix}}=\Delta H_{\mathrm{mix}}-T\Delta S_{\mathrm{mix}}$$

**FIGURE 8:**
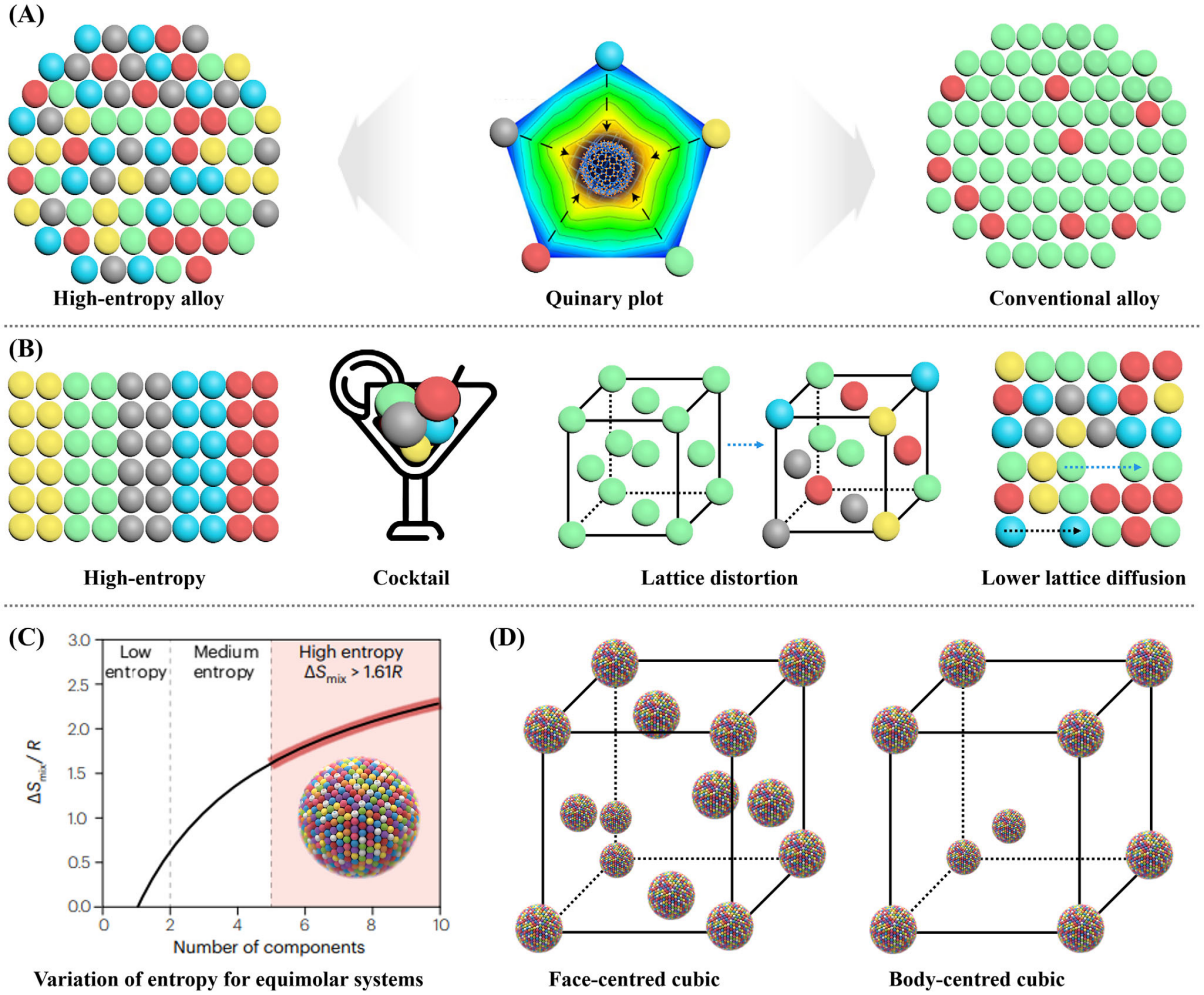
Fundamental characteristics of HEA NPs: (A) compositional comparison between HEA NPs and conventional alloy NPs; (B) distinctive physicochemical properties; (C) configurational entropy values; (D) prevalent crystal structures.

In this framework, the sign and magnitude of ∆*G*_mix_ are governed by the interplay between the enthalpy of ∆*H*_mix_ mixing and the entropic term ‐*T*∆*S*_mix_. A negative ∆*G*_mix_ signifies thermodynamic favorability toward a homogeneous single‐phase state characterized by random atomic distributions, whereas a positive value indicates a driving force for phase separation. For an ideal solution, the configurational entropy of mixing is expressed as:

$$\Delta S_{\mathrm{mix}}=-R\Sigma x_{i}\ln x_{i}$$

From this relation, it follows that ∆*S*_mix_ reaches maximum when all components are present in equimolar proportions. Conversely, ∆*H*_mix_ exhibits significant variation across phase‐segregated mixtures and ordered intermetallic compounds, giving rise to distinct critical temperatures at which the entropic contribution *T*∆*S*_mix_ surpasses the enthalpic contribution ∆*H*_mix_. In single‐phase solid solutions, ∆*H*_mix_ is typically negligible, as is the non‐configurational entropy. Consequently, at sufficiently elevated temperatures, configurational entropy emerges as the dominant factor governing phase stability. The incorporation of a greater number of components in near‐equimolar ratios substantially enhances the entropic contribution, thereby reducing the overall Gibbs free energy and promoting single‐phase formation. The above illustrated that their phase stability depends on the competition between mixing enthalpy and mixing entropy, while confining multiple elements with widely different properties in the same lattice is inherently energetically unfavorable. Statistics show that the enthalpy gain of the system drops sharply with increasing number of elements, approaching zero for quaternary systems [227]. Although this favors entropy stabilization, it also makes the screening of elements via traditional trial‐and‐error methods exceptionally time‐consuming. Moreover, good thermodynamic stability does not equate to ease of synthesis; the latter is also constrained by the specific formation pathway, and the situation becomes more complicated when non‐equiatomic designs are adopted for performance optimization. At the structural‐control level, synthesis often requires harsh conditions such as high temperature and high pressure to enhance the entropy term and overcome the high energy barrier for atomic interdiffusion [228]. As a result, conventional methods tend to prioritize achieving completely random atomic mixing, making it difficult to finely design the microstructure. The inability to effectively control key parameters such as crystal form, grain characteristics, exposed crystal faces, and various defects makes it hard to establish clear and consistent model systems, which constitutes a major bottleneck for in‐depth research and has also led to disagreements in the academic community on fundamental issues such as long/short range order and heterogeneity [229, 230]. Therefore, developing controllable synthesis methods is crucial for revealing the intrinsic properties of the materials. In recent years, thanks to advances in multiscale synthesis, characterization, and simulation techniques, preparation strategies for HEMs have seen significant progress [231, 232]. Their synthesis concepts can largely be categorized into two major types: thermodynamic control and kinetic control. Furthermore, quantitative techniques such as energy‐dispersive X‐ray spectroscopy (EDS) or inductively coupled plasma mass spectrometry (ICP‐MS) are employed for this analysis. Second, the material should form a single solid‐solution phase of fcc, bcc, or hcp, as evidenced by X‐ray diffraction (XRD) or selected‐area electron diffraction (SAED) patterns that display the characteristic signals of a simple crystal structure without extraneous peaks from secondary phases. Most critically, uniform distribution of all elements at the nanoscale must be confirmed, excluding elemental segregation or core‐shell formation [233]. This is typically achieved through direct visualization using high‐angle annular dark‐field scanning transmission electron microscopy (HAADF‐STEM) coupled with elemental mapping. Furthermore, observing distinctive properties arising from severe lattice distortion, such as exceptional catalytic activity or high thermal stability, provides additional support for the material's high‐entropy nature. Thus, the essential criteria for confirming HEAzyme are multi principal elemental composition, a single‐phase solid‐solution structure, and nanoscale elemental homogeneity [234]. To assist researchers in making accurate determinations, this section reviews recent advances in the validation of HEAzymes, focusing on evidence pertaining to composition, structure, and performance.

### 3.1 Characterization

#### 3.1.1 Compositional Characterization

Compositional characterization forms the fundamental basis for determining whether a nanozyme meets the definition of a high‐entropy system [235]. Its primary purpose is to verify that the material consists of multiple principal metal elements in near‐equip atomic proportions, with each element's content within the 5–35% range, thereby laying the groundwork for subsequent structural and property analysis. Characterization strategies are broadly divided into bulk analysis and micro‐scale distribution analysis. Bulk analysis, performed using techniques such as inductively coupled plasma mass spectrometry (ICP‐MS) or atomic emission spectroscopy (AES), provides the accurate average chemical composition of the material. Micro‐scale analysis primarily relies on energy‐dispersive X‐ray spectroscopy (EDS) coupled with scanning/transmission electron microscopy (SEM/TEM), enabling local composition analysis via point, line, and area scans. Notably, HAADF‐STEM combined with EDS mapping is crucial for visually confirming nanoscale elemental homogeneity, ruling out segregation or phase separation, and supporting the identification of a single‐phase solid solution [236, 237, 238]. For ultimate resolution, atom probe tomography (APT) can resolve 3D elemental distributions at the atomic scale, representing the definitive technique for probing solid‐solution randomness and microscopic segregation. The comprehensive significance of compositional characterization is multifold: (1) it rigorously tests compliance with the HECs definition, providing the primary criterion for material qualification; (2) based on the compositional data, key parameters such as mixing entropy and atomic size difference can be calculated, aiding in the thermodynamic and structural explanation of simple solid‐solution formation; (3) composition and its distribution are essential for establishing the composition‐structure‐property relationship and for interpreting the cocktail and lattice distortion effects; and (4) the results can feedback into and optimize the synthesis process [239, 240, 241]. Therefore, compositional characterization not only clarifies chemical constitution but also verifies the high‐entropy nature and elemental homogeneity of HEAzymes, providing necessary experimental support for revealing their unique structural features, performance, and intrinsic high‐entropy effects.

#### 3.1.2 Structural Characterization

As shown in Figure 9, structural characterization is critical for confirming the formation of a single‐phase solid solution, revealing lattice distortion features, and correlating these with the unique properties of HEAzymes. Its core objective is to validate the high‐entropy effect at the crystallographic level. Key characterization methods include: (1) X‐ray diffraction (XRD), used to identify the crystal structure type and verify single‐phase nature, where significant peak broadening serves as direct evidence of lattice distortion induced by the multicomponent system; (2) electron diffraction and high‐resolution transmission electron microscopy (HRTEM), which confirm single‐phase structure at the micro‐scale and enable direct observation of lattice distortions, strain fields, and high‐density defects; (3) synchrotron radiation and neutron diffraction, capable of precisely analyzing atomic occupancy, degree of order, and local structural distortions while facilitating in situ studies of dynamic structural evolution; and (4) atom probe tomography (APT), which allows direct 3D analysis of elemental occupancy at the atomic scale, identifying short‐range order or incipient phase separation [242, 243, 244]. The comprehensive significance of structural characterization lies in the following. First, rigorously verifying the single‐phase solid‐solution characteristic of HEAzymes, then providing quantitative experimental evidence for concepts such as severe lattice distortion, following establishing structure‐property correlations to clarify the relationship between single‐phase structure and properties such as chemical‐thermal stability, finally, revealing the influence of lattice distortions and defects on mechanical behavior and elucidating the role of local structure in regulating functions like catalytic activity and magnetic properties [245]. Furthermore, the results can guide the optimization of composition and synthesis processes to enhance phase stability and overall performance. In summary, structural characterization, spanning from macroscopic diffraction to atomic‐scale imaging, systematically reveals the single‐phase solid‐solution essence and severe lattice distortion characteristic of HEAzymes. These features are not only structural markers that distinguish them from traditional materials but also the micro‐origin of their exceptional stability, catalytic performance, and functional properties. Together with compositional analysis, structural characterization forms a complete scientific framework for understanding, designing, and applying HEAzymes.

**FIGURE 9:**
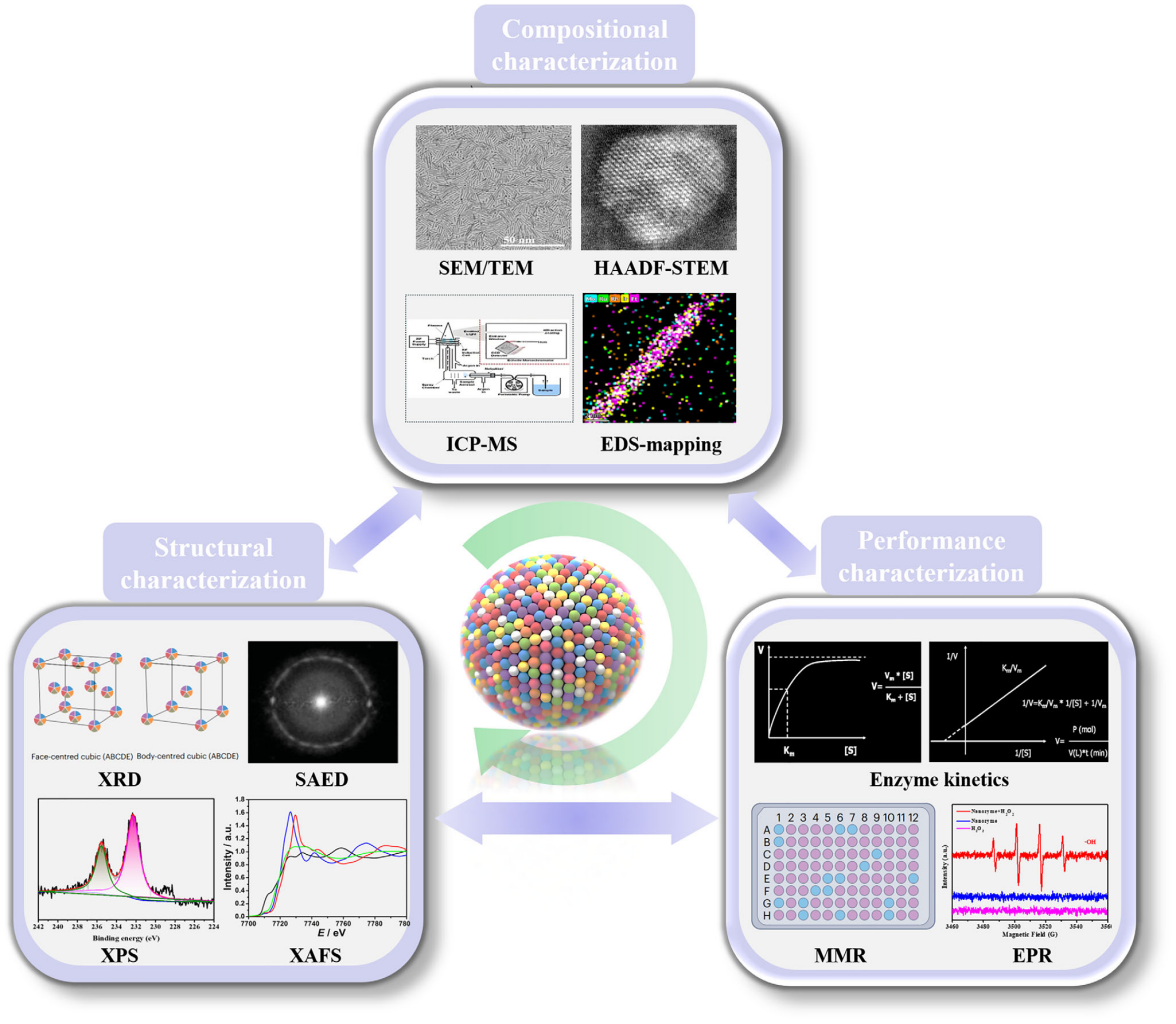
Determination of HEAzymes: general compositional characterization, structural characterization and performance characterization methods.

#### 3.1.3 Performance Characterization

Performance characterization aims to systematically evaluate the functional profile of HEAzymes and to validate their properties through distinctive and superior performance metrics [246, 247, 248]. This assessment extends beyond their core catalytic function as nanozymes to encompass the stability, robustness, and multifunctionality expected of HEMs [249]. The characterization covers three main areas: (1) Catalytic performance: evaluated through enzyme‐like kinetic analysis (e.g., determining Michaelis constant and maximum reaction rate), multienzyme activity profiling, and electrochemical testing. This verifies the multielement synergistic cocktail effect and correlates enhanced activity with structural features like lattice distortion. (2) Stability and durability: assessed via thermogravimetric analysis, high‐temperature in situ XRD, exposure to extreme pH/salinity/biomedical media, and cyclic catalytic tests. These studies reveal the slow diffusion effect and structural robustness inherent to high‐entropy systems, key to their practical application. (3) Functional application performance: demonstrated through antibacterial/antibiofilm assays, tumor catalytic therapy evaluations, and the construction of biosensing platforms [250, 251, 252, 253]. The overarching significance of performance characterization is threefold. First, it provides performance‐driven evidence for the value of high‐entropy characteristics, demonstrating superiority over traditional material systems in catalytic activity and stability. Second, it completes the logical chain of composition‐structure‐performance‐application, clarifying the material's functional efficacy and mechanism of action. Third, performance data offer critical feedback for inversely guiding component design and synthesis optimization, thereby expanding potential application scenarios. In conclusion, performance characterization serves as the ultimate benchmark for examining the attributes and enzyme‐mimetic activities of HEAzymes. It provides the scientific basis for translating theoretical advantages into practical applications, and together with compositional and structural characterization, constitutes a complete paradigm for systematic research in this field.

### 3.2 Catalytic Characteristics and Mechanisms of HEAzymes

As an emerging frontier in nanozyme research, HEAzymes are formed by incorporating five or more principal elements in equimolar or near‐equimolar ratios into a single nanocrystalline lattice. Their unique high‐entropy and cocktail effects substantially broaden the compositional space and regulatory dimensions of nanozyme performance [254]. In contrast to conventional single‐ or dual‐element nanozymes, HEAzymes not only preserve the fundamental ability to mimic natural enzymatic functions [255]. owing to synergistic multielement active centers and tunable electronic structures, exhibit a wider spectrum of enzyme‐like activities, higher catalytic efficiency, and more sophisticated cascade reaction capabilities. Therefore, systematically categorizing their types of enzyme‐like activities, analyzing their distinctive catalytic properties, and elucidating the underlying mechanisms rooted in multielement synergy are essential for the rational design and functional application of these materials. Accordingly, this section will focus on three key aspects: the types of enzyme‐like catalytic activities, catalytic characteristics, and underlying mechanisms.

HEAzymes are capable of mimicking a variety of natural enzyme activities. Major types include. POD catalyzes the oxidation of substrates in the presence of H_2_O_2_ generate ROS, whereas oxidase directly uses oxygen to oxidize substrates; catalase decomposes hydrogen peroxide into water and oxygen, superoxide dismutase catalyzes the disproportionation of superoxide anions, and hydrolase promotes the hydrolysis of chemical bonds such as esters and amides [256]. Other reported activities include haloperoxidase, lyase, and isomerase. A defining feature of HEAzymes is their multienzyme mimicry, enabling a single nanozyme to concurrently perform multiple enzymatic functions [257]. It should be noted, however, that certain enzymatic activities, such as those of translocases, transferases, and ligases, have not yet been successfully replicated by HEAzymes. The enzyme‐like catalysis of HEAzymes exhibits several notable characteristics. They display multienzyme mimicry, allowing a single nanozyme to simulate the functions of several natural enzymes, such as POD, oxidase, CAT, and SOD, thereby catalyzing diverse biochemical reactions (Figure 10). Moreover, their catalytic activity and selectivity can be precisely tailored through rational regulation of elemental composition, crystal phase, morphology, and surface structure [214]. Compared to natural enzymes, HEAzymes demonstrate superior stability, maintaining activity under harsh conditions such as high temperatures and extreme pH, alongside advantages in cost‐effectiveness and scalable production. Importantly, their inherent multienzyme functionality enables them to serve as integrated nanocatalytic platforms that drive efficient multistep cascade reactions, facilitating continuous substrate conversion and significantly enhancing overall catalytic efficiency [258]. These attributes collectively underscore the strong potential of HEAzymes in biosensing, therapeutic applications, and environmental remediation. This subsection has summarized and discussed the key catalytic characteristics of HEAzymes.

**FIGURE 10:**
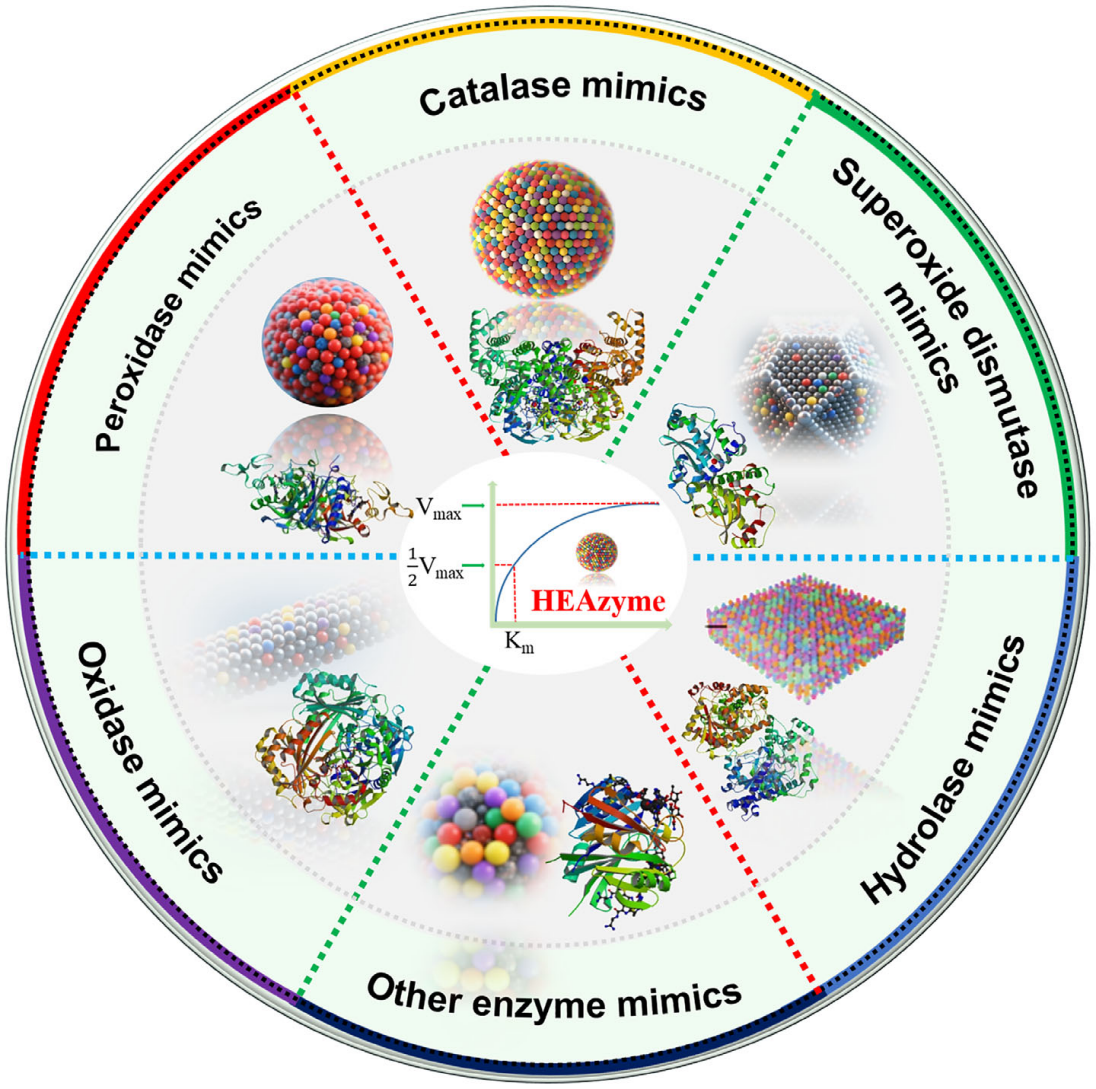
Catalytic characteristics and mechanisms of HEAzymes.

The catalytic mechanisms of HEAzymes are complex and diverse, which can be systematically categorized into three types: redox catalysis, hydrolytic catalysis, and activity regulation mechanisms [259, 260]. In terms of redox catalysis, POD‐like reactions primarily generate highly reactive hydroxyl radicals (^•^OH) through Fenton‐type processes or directly catalyze substrate oxidation via non‐Fenton electron transfer pathways [261]. Meanwhile, oxidase‐like activity relies on ROS‐mediated pathways or electron transfer mechanisms to activate molecular oxygen and complete the catalytic cycle [262]. Regarding hydrolytic catalysis, the mechanisms are highly biomimetic [263]. On one hand, they involve constructing enzyme‐like metal active centers to achieve synergistic catalysis. On the other hand, they utilize simulated amino acid microenvironments to facilitate proton transfer and promote substrate hydrolysis. At the level of activity regulation, the underlying mechanisms include precise modulation of the local electronic structure at the atomic level to optimize catalytic efficiency, as well as leveraging multielement synergistic effects such as the cocktail effect and hydroxyl spillover phenomenon to significantly enhance overall catalytic performance. However, it is evident that the cocktail effect has not yet been established as a genuine catalytic mechanism. Whether the enhanced performance stems from d‑band‑center modulation, lattice‑distortion/strain effects, multi active site synergy, or simply the superposition of individual elemental contributions remains unresolved.

Compared with conventional multimetallic nanozymes, such as binary or ternary systems, the catalytic advantages of HEAzymes do not arise from merely increasing the number of constituent elements, but instead originate from intrinsic entropy‐driven effects. At the electronic‐structure level, the restricted intermetallic d–d coupling in conventional systems can only induce localized charge transfer; in contrast, the high‐entropy state features strong multielement d‐orbital hybridization and mutual electronic modulation, resulting in a significant increase in the density of states near the Fermi level, a systematic shift of the d‐band center, and the emergence of the cocktail effect [264, 265, 266]. This permits continuous tuning of adsorption energies at catalytic interfaces through compositional adjustment, bringing them closer to the Sabatier optimum. Structurally, lattice distortion in conventional alloys is limited to atomic‐size mismatches among a few elements. However, the cumulative differences in atomic radii, bond energies, and electronegativities across multiple constituents induce severe, pervasive lattice distortion, generating a structurally non‐equilibrium state in HEAzymes [267]. This markedly increases the density of surface defects, vacancies, and under‐coordinated sites, thereby effectively reducing activation energies for substrate adsorption and conversion. In terms of stability, conventional multimetallic nanozymes are susceptible to elemental segregation, phase separation, and sintering during catalytic operation; conversely, HEAzymes with configurational entropy exceeding 1.5*R* are thermodynamically locked into a single‐phase solid solution, whilst the sluggish diffusion effect substantially suppresses atomic migration and grain growth, imparting exceptional catalytic durability. Regarding catalytic functionality, the heterogeneous distribution of active sites in HEAzymes not only enables simultaneous mimicry of diverse natural enzymes, but also facilitates complex multienzyme cascade reactions and the modulation of reactive oxygen and nitrogen species steady‐state levels [268]. In essence, the catalytic superiority of HEAzymes derives from the synergy of entropy stabilization, severe lattice distortion, and multielement d‐electron coupling, conferring upon them the capacity to surpass conventional multimetallic nanozymes in activity, selectivity, stability, and resilience under extreme conditions.

## 4 Applications of HEAzyme

HEAzyme which integrate five or more elements into a single solid‐solution phase at the atomic scale, exhibit remarkable performance potential surpassing that of conventional materials, owing to their unique high‐entropy, lattice distortion, and sluggish diffusion effects [269, 270, 271]. The underlying mechanisms can be attributed to several key factors: the high configurational entropy effectively reduces the Gibbs free energy of the system, thereby enhancing thermodynamic stability. Severe lattice distortion caused by atomic size mismatch modulates the electronic structure of active sites, optimizing the adsorption behavior of reaction intermediates and enhancing intrinsic catalytic activity [272, 273, 274]. In addition, the multicomponent synergy enables directional control over material properties, allowing for performance tailoring on demand, thus HEMs demonstrate promising applications across a range of cutting‐edge technological fields [275]. For example, sub‐nanometer HEA catalysts significantly reduce the overpotential for water electrolysis while improving long‐term durability. Furthermore, high‐entropy nanocatalysts enable highly efficient electrocatalytic degradation of pollutants with simultaneous production of high‐purity hydrogen. These materials further offer innovative solutions of fuel cell electrodes, antibacterial therapy, and flexible electronics, addressing critical challenges related to stability under harsh conditions, bioactivity, and mechanical compliance [276, 277, 278, 279]. The compositional space and tunable properties provide a versatile platform for the design and development of advanced functional materials. In this section, we systematically review recent advances in versatile applications of HEAzyme, focusing on their applications in antioxidant strategies, disease treatment, biosensing, energy‐related catalysis, and other emerging areas (Figure 11). The aim is to offer valuable insights for the targeted design and expanded application of these innovative materials.

**FIGURE 11:**
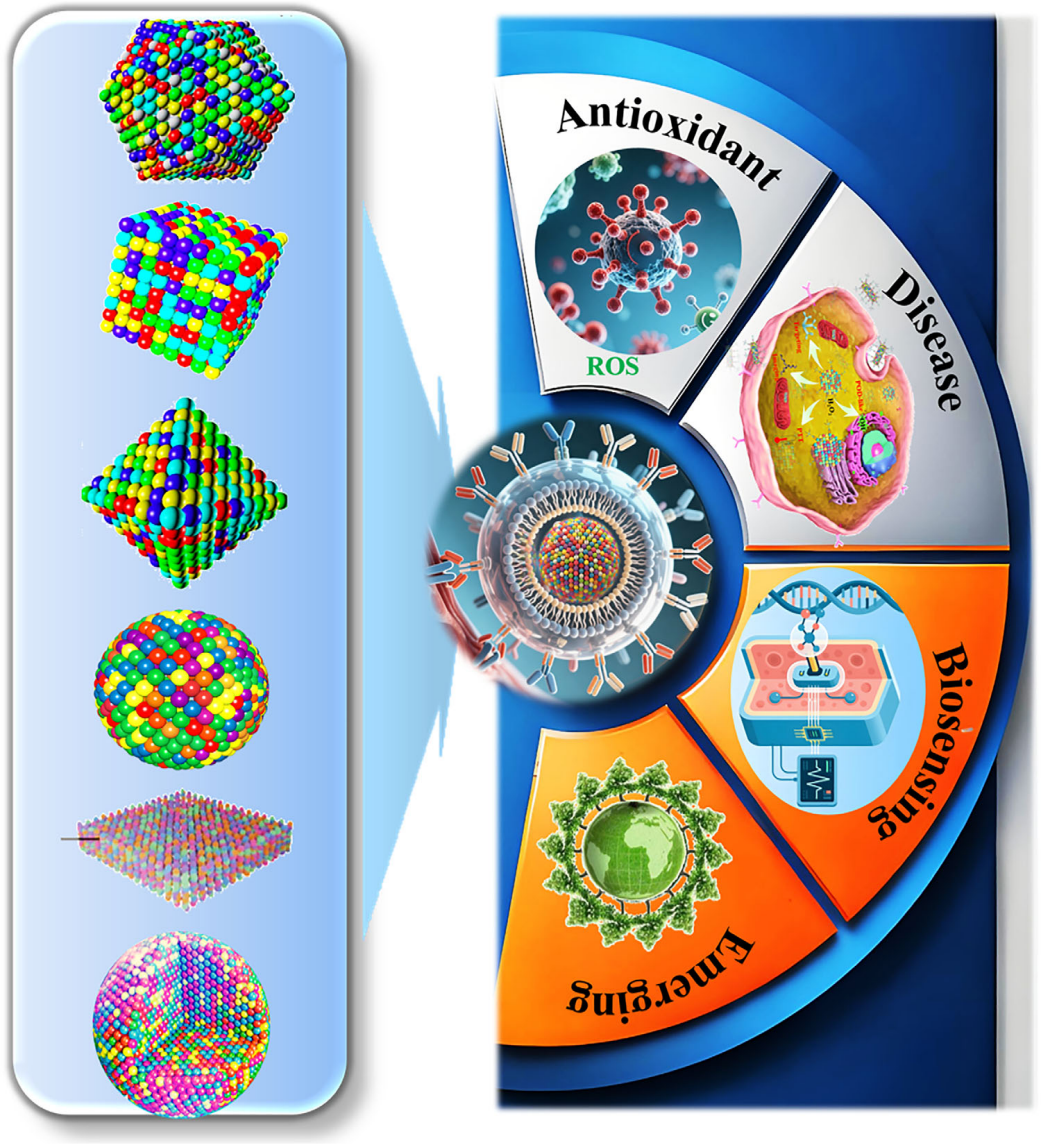
Versatile applications of HEAzyme: antioxidant, disease treatment, biosensing, energy‐related catalysis, and other emerging areas.

### 4.1 HEAzymes for Antioxidant

HEAzymes demonstrate significant potential in antioxidative research owing to their multielement synergistic effects and tunable electronic structures [280, 281, 282]. Their antioxidative mechanisms extend beyond the direct catalytic degradation of reactive species to encompass the regulation of oxidative stress‐related cellular pathways, such as ferroptosis [283]. Moreover, through material design strategies such as defect engineering and cation doping, the catalytic activity and electron transfer behavior of HEAzymes can be precisely modulated, allowing for synergistic enhancement or even dynamic switching of their antioxidative functions [284]. This versatility positions HEAzymes as a highly programmable platform for intervening in oxidative stress‐related diseases. In recent years, research in this area has progressed rapidly, and this section systematically reviews the latest advances in the antioxidant applications of HEAzymes.

Among these advances, Huo et al. developed an amorphous D‐handed PtPd_2_CuFe HEAzyme with chiral‐dependent senolytic activity (Figure 12A) [285]. Using multi‐omics analyses, the study revealed that the senescent microenvironment induces the enrichment of copper and iron atoms in low‐oxidation states. This enrichment disrupts mitochondrial iron‐thiol clusters and lipoic acid transferase while promoting the oxidation of unsaturated fatty acids, thereby triggering cuproptosis and ferroptosis. Notably, the nanozyme exhibited concentration‐dependent anti‐aging effects, demonstrating broad‐spectrum therapeutic efficacy against atherosclerosis even at ultralow doses. Biosafety indicators also suggest that this HEAzyme holds great promise. As shown in Figure 12B, He et al. reported a PtIrRuRhCu HEAzyme featuring triple enzyme‐like activities, such as POD, CAT, and SOD for the treatment of tendinopathy [286]. Mechanistic investigations revealed that the nanozyme alleviates oxidative stress by upregulating the PGAM5/FUNDC1/GPX4 axis, thereby restoring mitochondrial autophagy. This work provides a nanozyme‐based strategy for antioxidant intervention in tendinopathy, highlighting the therapeutic potential of multienzyme mimicking HEAzymes in inflammatory musculoskeletal disorders. Complementing these studies, Lee et al. synthesized ultra‐small Pt group HEAzymes with tunable morphologies [68]. Compared with their monometallic counterparts, these HEA nanozymes exhibited markedly enhanced POD‐like activity and effectively mimicked the cascade regulation of reactive oxygen and nitrogen species (RONS), encompassing SOD‐like, catalase‐like, ^•^OH antioxidant capacity (HORAC), and nitric oxide scavenging activities. As a result, they achieved superior anti‐inflammatory efficacy through intrinsic activity enhancement and cascade reaction mechanisms, underscoring the advantage of high‐entropy configurations in amplifying antioxidant functions. Beyond the above examples, Cheng et al. designed a PtPdRuRhIr HEAzyme featuring a modulated d‐band center and pH‐switchable ROS biocatalysis (Figure 12C) [287]. The unique electronic structure of this material stabilizes the d‐band center and optimizes oxygen binding, enabling antioxidase‐like functions under physiological conditions while switching to POD‐like activity in acidic infectious environments. This pH‐adaptable platform simultaneously supports tissue regeneration and biofilm disruption, offering a smart strategy for the stage‐specific treatment of periodontitis and other biofilm‐associated inflammatory diseases. The above results illustrated that HEAzymes achieve potent antioxidant effects through diverse design strategies and mechanistic pathways. These strategies and pathways range from chiral‐dependent senolysis and mitochondrial autophagy regulation to cascade RONS scavenging and pH‐switchable biocatalysis. Futherore, the HEAzymes also showed satisfactory biocompatibility and biosafety [288]. This study suggests that HEAzymes hold promise as a next‐generation therapeutic platform for diseases associated with oxidative stress.

**FIGURE 12:**
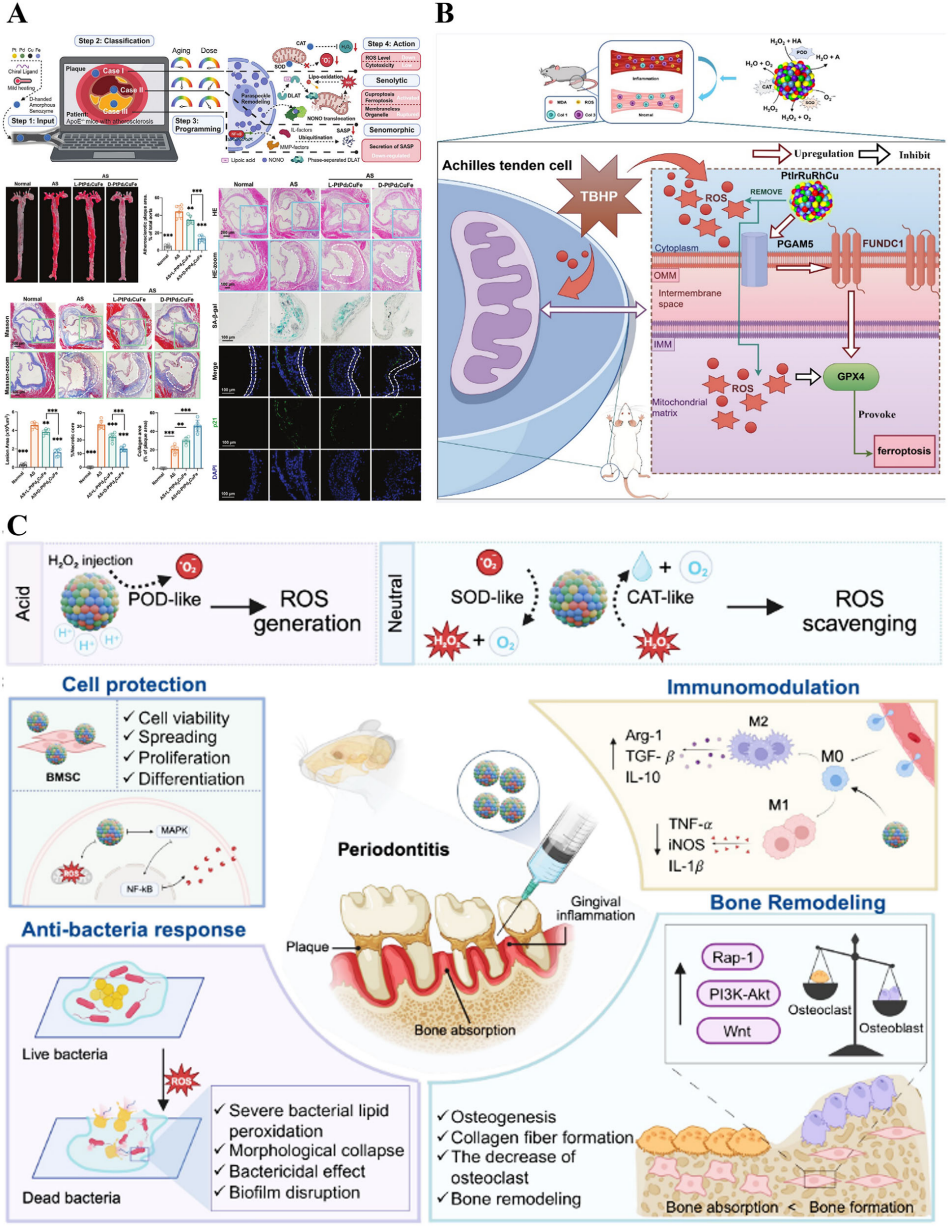
HEAzymes for antioxidant: (A) HEAzyme with peroxide and SOD‐like activity for antioxidant in combat senescence. Reprinted with permission from Ref. [285] Copyright 2024, Wiley‐VCH. (B) HEAzymes as ROS biocatalyst for antioxidant in tendinopathy. Reprinted with permission from Ref [286]. Copyright 2025, Springer Nature. (C) HEAzyme as pH‐controllable ROS biocatalysis for stage‐specific antioxidant in inflammatory and infectious oral diseases. Reprinted with permission from Ref. [287] Copyright 2025, Wiley‐VCH.

### 4.2 HEAzyme for Disease Treatment

HEAzymes demonstrate remarkable advantages over conventional nanozymes in disease treatment, leveraging their unique multielement active sites and tunable electronic structures to offer innovative strategies for managing various refractory diseases. In anti‐infection therapy, HEAzymes exhibit enhanced POD‐like activity, efficiently converting endogenous H_2_O_2_ into highly toxic free radicals, with multielement synergistic effects significantly improving bactericidal efficiency and presenting a novel approach to addressing antibiotic resistance [289, 290, 291]. Their exceptional multienzyme mimicking capability enables simultaneous clearance of diverse ROS, effectively breaking the cycle of oxidative stress, and when integrated with biomaterials, they form smart regenerative systems that substantially accelerate tissue repair [292]. In oncology, HEAzymes can intelligently respond to the tumor microenvironment, synergistically eliminating cancer cells through multiple mechanisms such as cuproptosis induction and glutamine metabolism disruption, while further functionalization via surface modification enables targeted delivery, highlighting their potential for precision therapy. For complex neurological inflammatory diseases, HEAzymes achieve effective blockade of the oxidative‐inflammatory cascade by precisely regulating key pathways including pyroptosis. Owing to their unique combination of multienzyme‐like catalytic activities, tunable electronic structures, and synergistic physicochemical properties, HEAzymes have garnered particular attention in tumor therapy, offering a versatile platform for tumor‐specific interventions through catalytic ROS generation, photothermal conversion, biorthogonal catalysis, and immunomodulation [293, 294, 295]. These groundbreaking advances demonstrate that HEAzymes not only expand the application boundaries of nanozymes in biomedicine but also provide a novel material platform for developing next‐generation intelligent diagnostic and therapeutic systems. Based on this progress, this section systematically reviews recent advances in the application of HEAzymes for disease treatment, with a focus on cancer therapy, highlighting the diversity of design strategies and therapeutic mechanisms.

As shown in Figure 13A, we fabricated PtPdRuRhIr ultra‐small HEA NPs (US‐HEA NPs) as bifunctional nanoplatforms for efficient tumor therapy [45]. These NPs were synthesized via a universal metal‐ligand cross‐linking strategy based on the aldol condensation of organometallics, a simple and scalable approach. The resulting US‐HEA NPs exhibited excellent POD‐like activity, enabling the catalytic conversion of endogenous hydrogen peroxide into highly toxic ^•^OH. In addition, they demonstrated a high photothermal conversion effect under 808 nm NIR irradiation. Both in vitro and in vivo experiments confirmed that the synergistic combination of POD‐like activity and photothermal action effectively ablated cancer cells and suppressed tumor growth. The ICP‐MS analysis revealed that the metal components of the HEAzymes degrade within the body, and metal ions were detected in major organs such as the liver. However, owing to the extremely small size of the HEAzymes, the concentrations of these metal ions remain within safe limits. This work not only provides a new synthetic route for HEA NPs but also establishes a foundation for their biomedical applications. Building upon this, we further developed an atomic level HEAzymes system capable of endogenous targeted catalysis for enhanced tumor photothermal therapy. A RuRhPtIrMo sub‐nanometer HEAzyme with a thickness of approximately 8–10 atoms was first prepared via a reduction‐diffusion method (Figure 13B) [210]. The *sn*HEAzyme was subsequently functionalized with the targeting ligand DSPE‐PEG2000‐cRGD and the imaging agent Cy7 to construct the *sn*HEAzyme@DSPE‐PEG2000‐cRGD@Cy7 system. This HEAzyme exhibited excellent POD‐like activity and strong absorbance in the NIR region. Under NIR irradiation, it demonstrated efficient photothermal conversion and enhanced ROS generation. In vitro and in vivo studies confirmed effective tumor targeting and penetration, leading to robust antitumor efficacy with good biosafety. This work offers new perspectives on the fabrication of sub‐nanometer HEAzymes and their biomedical applications.

**FIGURE 13:**
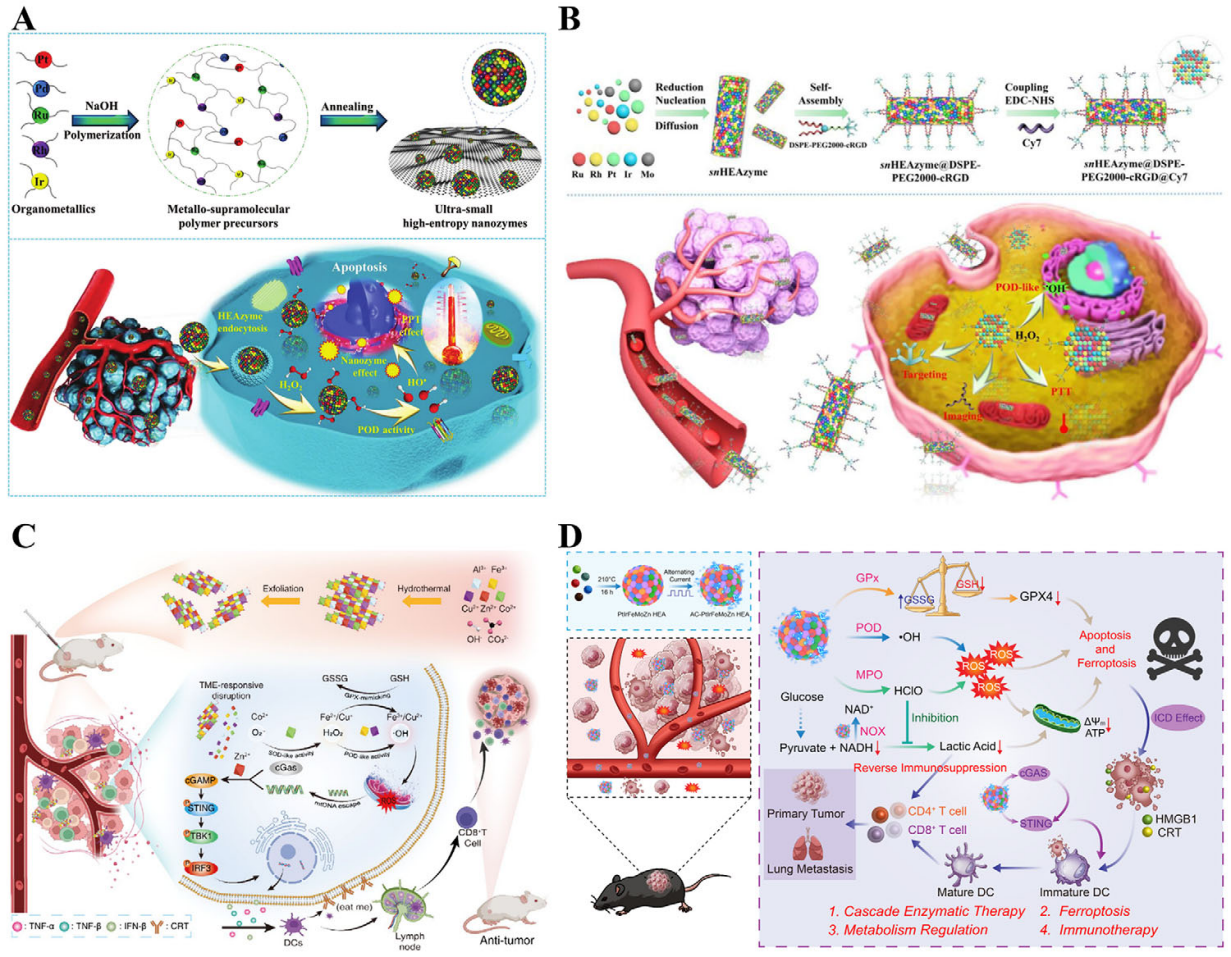
HEAzymes for disease treatment: (A) US‐HEA NPs as efficient nanozyme for enhancing tumor photothermal therapy. Reprinted with permission from Ref. [45] Copyright 2023, Wiley‐VCH. (B) Atomic‐level HEAzymes for remarkable endogenous targeted tumor catalysis therapy. Reprinted with permission from Ref. [210] Copyright 2025, Wiley‐VCH. (C) 2D nanosheet‐like HEAzymes for chemodynamic and immune therapy. Reprinted with permission from Ref. [302] Copyright 2025, American Chemical Society. (D) HEAzymes regulate metabolism for tumor immunotherapy. Reprinted with permission from Ref. [303] Copyright 2025, Springer Nature.

Medium‐entropy nanozymes (MEzymes) typically comprising three to four metallic elements, have attracted growing interest in biomedicine owing to their multielement synergistic effects and tunable electronic structures. Rational design strategies have progressively expanded their applications in disease therapy, bioimaging, and clinical diagnosis. our group developed an ultrasmall MEzyme system (MEzyme@HCCM) camouflaged with homologous cancer cell membranes [296]. The RuPtIrMo nanozyme core, prepared via a metal‐ligand cross‐linking strategy, was coated with homologous membranes to enable specific tumor recognition and targeted delivery. The nanozyme exhibited POD‐like activity, catalyzing endogenous H_2_O_2_ into cytotoxic ^•^OH. In vivo studies demonstrated tumor growth inhibition exceeding 60%. Proteomic analysis further revealed ROS‐mediated signaling pathways involving DNA damage, proliferation suppression, apoptosis induction, and autophagy activation. This work establishes a new paradigm for the fabrication of MEzymes and expands their biomedical applications. Recently, Wang et al. constructed a microglial membrane‐wrapped single‐atom nanozyme by integrating single‐atom catalysts with medium‐entropy alloys [297]. Biomimetic membrane coating enabled blood‐brain barrier penetration and accumulation in neuroinflammatory regions following intracerebral hemorrhage, allowing dual regulation of ROS and the inflammatory microenvironment. The nanozyme displayed ^•^OH scavenging and SOD/CAT‐mimicking activities, repolarizing microglia from the pro‐inflammatory M1 to the anti‐inflammatory M2 phenotype in vitro and in vivo. This ameliorated neurofunctional deficits and spatial memory impairments while improving survival in model animals, offering a reference for nanocatalytic intracerebral hemorrhage therapy. For bioimaging and biosensing, Ju et al. designed a MEzymes with a trinuclear Prussian blue analogue structure to address the challenge of transient chemiluminescence (CL) [298]. Rapid intermetallic electron transfer within the framework enabled recycling regeneration of the Co(II) catalytic center, overcoming conventional nanozyme limitations in CL reactions. The material achieved stable CL emission enhancement exceeding two orders of magnitude over one hour, transforming the signal from a transient flash to a sustained glow. Combined experimental and DFT studies elucidated the CL enhancement mechanism and active valence cycling, with practical demonstration via bacterial motility imaging. This work provides a design strategy for persistent CL systems and expands nanozyme applications in bioanalysis. Following, Yamauchi et al. designed mesoporous MEzymes to address the catalytic limitations of platinum‐based nanozymes in peroxidase‐mimicking reactions [299]. Excessive p‐d orbital hybridization between Pt 5d and O 2p orbitals typically causes oxygen poisoning and high reaction energy barriers. Multielement synergy in the MEzymes system lowered Pt 5d orbital energy, reduced antibonding levels, and weakened p‐d hybridization, thereby decreasing energy barriers and suppressing oxygenated intermediate adsorption (^*^OH_2_/^*^OH). The mesoporous architecture enhanced active site accessibility, yielding a POD specific activity of 6.58‐fold and 507‐fold higher than mesoporous Pt and non‐porous MEzymes, respectively. The derived immunoassay for prostate‐specific antigen achieved a detection limit of 1.20 pg L^−1^. This work demonstrates an orbital engineering approach to overcoming noble‐metal nanozyme limitations for advanced biosensing and diagnostics.

The fundamental distinction between MEzymes and HEAzymes lies in their configurational entropy values. MEzymes typically exhibit configurational entropy in the range of 1.0–1.5*R* and are composed of 3–4 principal elements, whereas HEAzymes possess entropy values greater than 1.5*R* and generally comprise 5 or more principal elements. This compositional divergence gives rise to markedly different structural and catalytic characteristics. HEAzymes benefit from severe lattice distortion and the multielement synergistic cocktail effect, which collectively generate heterogeneous local coordination environments and diverse active sites. Consequently, they typically exhibit enhanced catalytic versatility and structural stability [300]. By contrast, the relatively streamlined elemental palette of MEzymes facilitates more precise compositional tuning, enabling targeted modulation of electronic structures and local atomic environments [297]. This renders MEzymes particularly advantageous for applications demanding high catalytic specificity.

Beyond direct catalytic and photothermal effects, HEAzymes have been exploited for biorthogonal catalysis and tumor microenvironment (TME) modulation. Qu et al. engineered a nuclear‐targeted biorthogonal system based on high‐entropy principles, in which the high‐entropy coordination environment stabilized palladium catalysts and prevented their deactivation [301]. Notably, the system exhibited intrinsic GP_X_‐like activity, depleting intracellular glutathione and further protecting the catalyst. This platform enabled direct drug synthesis within the nucleus, representing a novel paradigm for intracellular catalysis. As shown in Figure 13C, Kim et al. developed high‐entropy 2D layered double hydroxide (HE‐LDH) nanoplatforms that respond to the TME by sequentially releasing metal ions to exhibit superoxide dismutase (SOD), POD, and GPX‐like activities [302]. This cascade enzymatic activity converted tumor metabolites into cytotoxic ROS while alleviating hypoxia, enabling robust nanozyme‐initiated chemodynamic therapy. Furthermore, the release of Zn^2+^ activated the cGAS‐STING pathway, triggering innate immune responses and synergistic antitumor immunity. This work highlights the potential of HEAzymes to integrate chemodynamic and immunotherapeutic modalities. Complementing this, Hu et al. developed a PtIrFeMoZn HEAzyme via a simple hydrothermal method, demonstrating that alternating current pretreatment significantly enhanced its therapeutic efficacy (Figure 13D) [303]. The AC‐treated nanozyme exhibited synergistic POD, myeloperoxidase (MPO), and GPX‐like activities, generating a robust ROS storm while concurrently activating immunogenic cell death and the cGAS‐ STING pathway. Moreover, it disrupted glycolysis homeostasis by depleting NADH and generating hypochlorous acid, thereby augmenting both cell death and immune responses. This multipronged strategy exemplifies the potential of HEAzymes to orchestrate combined apoptotic, ferroptotic, and immunogenic antitumor effects. The rational design of HEAzymes has been further advanced by data‐driven approaches for multimodal therapeutic strategies [304, 305, 306, 307]. Zhang et al. employed a machine learning assisted framework to identify key metal elements for enhanced multicatalytic activity [262]. Guided by machine learning predictions, a PtFeMoCoNiRu HEA sub‐nanowire was synthesized, exhibiting exceptional ROS generation upon ultrasound activation. The resulting oxidative stress induced DNA damage and activated the cGAS‐STING pathway, leading to PANoptosis. This work demonstrates the power of machine learning‐guided strategies in accelerating the discovery of multicatalytic HEA nanomaterials. These studies illustrate the remarkable versatility of HEAzymes in tumor therapy, encompassing bifunctional photothermal catalytic platforms, sub‐nanometer engineered systems, biorthogonal catalysis, TME‐responsive chemodynamic immunotherapy, and machine learning‐guided rational design. The convergence of multiple enzymatic activities, tunable electronic structures, and synergistic physicochemical properties positions HEAzymes as a powerful and programmable platform for next‐generation cancer therapeutics. Although HEAzymes hold considerable promise for biomedical applications, their remains outstanding biosafety concerns. Because these nanomaterials typically comprise five or more metallic elements, their overall toxicity cannot be readily extrapolated from the toxicological profiles of individual components; the coexistence of multiple elements may instead give rise to unpredictable synergistic or antagonistic effects, accompanied by the activation of immune and inflammatory pathways, disruption of ROS homeostasis, and cumulative injury to organs such as the liver, kidneys, and spleen. Moreover, the potential risks associated with long‐term, low‐dose exposure remain insufficiently characterized. Compounding these concerns, in vivo microenvironments of acidic lesions, elevated glutathione levels, lysosomal compartments, and the protein corona will drive the heterogeneous leaching of metal components at differential rates. Such non‐uniform ion release can perturb the homeostasis of essential metal ions while compromising the structural integrity of catalytic active sites, leading to a progressive loss of catalytic activity and even secondary toxicity. In parallel, particle size and surface charge largely determine whether nanozymes undergo renal clearance or are sequestered by the liver and spleen, whereas blood‐flow shear, high ionic strength, proteolytic degradation, and redox fluctuations may further induce surface‐ligand desorption, elemental segregation, valence‐state transitions, and lattice etching, thereby accelerating the time‐dependent decay of enzyme‐mimetic activity and altering their absorption, distribution, metabolism, and excretion profiles.

### 4.3 HEAzyme for Biosensing

As an emerging class of bioinspired catalytic materials, HEAzymes exhibit unique application potential in biosensing, owing to their synergistic multielement active sites and tunable electronic structures [308]. Compared with natural enzymes, HEAzymes overcome intrinsic limitations such as poor stability, high production costs, and stringent storage requirements. Furthermore, by inheriting the advantageous features of nanomaterials, including large specific surface areas and ease of surface functionalization [309]. They offer a versatile platform for constructing high‐performance, multifunctional biosensing systems [310]. Recent advances have demonstrated their broad utility across diverse sensing scenarios: in environmental monitoring, HEAzymes enable simultaneous pollutant detection and degradation; in disease diagnostics, integration with portable devices significantly enhances point‐of‐care testing sensitivity; in complex sample analysis, they allow discriminative detection of multiple biomarkers using a single sensing unit; and in molecular imaging, logic‐gate‐controlled designs facilitate highly specific responses to particular disease markers [311, 312, 313]. Despite challenges related to catalytic efficiency, mechanistic understanding, standardization, and biosafety assessment, the exceptional stability, signal amplification capabilities, and intelligent detection features of HEAzymes position them as promising candidates for next‐generation biosensing technologies [314]. This section systematically reviews recent progress in the application of HEAzymes for biosensing, providing insights to guide future research and development.

HEAzymes have been extensively explored for the ultrasensitive detection of biomarkers [315]. For example, a 2D HEA nanosheet (HEA NS)‐based electrochemical aptasensor was developed for the detection of pro‐gastrin‐releasing peptide (ProGRP) [316]. Functionalized with polydopamine, the nanosheets provided abundant active sites and enhanced biocompatibility, enabling a broad detection range from 10 pg mL^−1^ to 100 µg mL^−1^ with a limit of detection (LOD) of 0.874 pg mL^−1^. In another study, HEA NPs integrated with iron‐based metal‐organic frameworks (Fe‐MOFs) were used to construct an electrochemical aptasensor for carcinoembryonic antigen (CEA) [317], achieving an exceptionally low LOD of 0.115 fg mL^−1^. For myoglobin detection, a split‐type photoelectrochemical (PEC) aptasensor was designed using Z‐scheme WO_3_/ZnIn_2_S_4_ heterostructures as the photoactive substrate and ultrathin PtCoFeRuMo HEA nanowires (HEA NWs) as signal amplifiers [318], resulting in an LOD of 0.96 fg mL^−1^. Furthermore, as shown in Figure 14A, a rational design strategy involving the substitution of Ru for Fe in FeMnCuCoNi HEAzymes yielded RuMnCuCoNi with a sevenfold increase in POD‐like activity [319]. This enhanced activity, attributed to optimized p‐d orbital hybridization, enabled the construction of an on‐site detection platform for the gastric cancer biomarkers D‑proline and D‑alanine with exceptional sensitivity and selectivity. HEMs have also been applied to detect biomarkers associated with inflammation and neurological function. For the non‐invasive diagnosis of inflammatory bowel disease, an electrochemical aptasensor based on HEANSs@AAs was developed for precise quantification of calprotectin [320]. This work demonstrating high stability, reproducibility, and acceptable recovery rates in human serum samples. For dopamine (DA) detection, an FeCuAgCeGd HEAzyme was synthesized, exhibiting POD‐like activity that catalyzes the generation of ^•^OH to oxidize 3,3,5,5‐tetramethylbenzidine [321]. The colorimetric sensor, based on the ^•^OH‐scavenging effect of DA, achieved an LOD of 0.77 µm, while a point‐of‐care testing (POCT) platform enabled on‐site visual detection with an LOD of 5.69 µm. In addition, HEMs have demonstrated significant potential in non‐invasive disease diagnosis through the detection of exhaled gas biomarkers. As shown in Figure 14B, Tang et al. developed a platform for exhaled H_2_S detection using nano‐high‐entropy Prussian blue frames based on nanozyme poisoning [322]. Integrated into a polyethylene microtube, the sensor achieved an LOD of 0.05 ppm with high specificity and stability, correlating well with gold‐standard methods and enabling on‐site testing for asthma and chronic obstructive pulmonary disease. To address the cost and scarcity of noble metals, a non‐noble MnFeCoNiCu HEAs was developed to enhance SnO_2_ for acetone detection [323]. The HEA‐SnO_2_ sensor exhibited high sensitivity, a low LOD of 30 ppb, and fast response/recovery times, offering a sustainable design strategy for advanced sensing materials.

**FIGURE 14:**
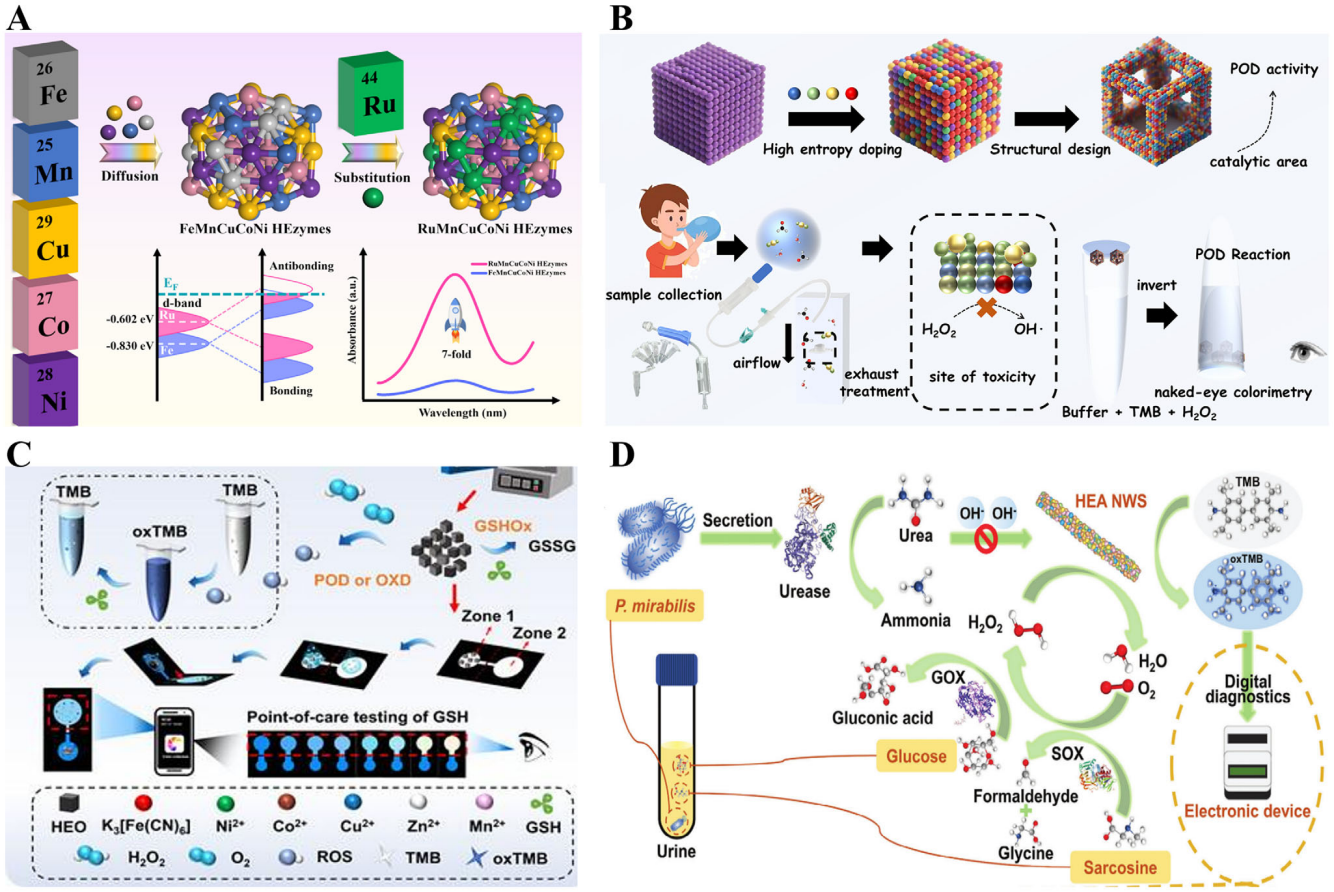
HEAzymes for biosensing: (A) Sensitive point‐of‐care detection of gastric cancer biomarkers. Reprinted with permission from Ref. [319] Copyright 2025, American Chemical Society. (B) Ultralow‐cost and rapid detection of exhaled H_2_S. Reprinted with permission from Ref. [322] Copyright 2025, American Chemical Society. (C) HEAzymes for cascaded glutathione detection and point‐of‐care testing. Reprinted with permission from Ref. [330] Copyright 2025, Wiley‐VCH. (D) Digital colorimetric monitoring of glucose, sarcosine, and *P. mirabilis* in urine. Reprinted with permission from Ref. [332] Copyright 2024, Wiley‐VCH.

The unique properties of HEMs have been leveraged to detect various contaminants in food and the environment. For tetracycline (TC) detection, a high‐entropy metal‐organic framework was constructed, featuring a microporous architecture that provided abundant binding sites for TC [324]. The sensor, which integrated sample pretreatment and detection, showed a linear range of 50–1000 nm and an LOD of 10.26 nm. Similarly, a high‐entropy layered double hydroxide (HE‐LDH, Fe_0.45_Co_0.45_Ni_0.45_Cu_0.45_Zn_0.45_‐LDH) with superior POD‐like activity was used to establish a colorimetric method for TC [325]. This work achieving LODs of 25.6 nm and 32.2 nm in solution and paper‐based modes, respectively. For chloramphenicol, a PEC aptasensor was developed using a multi interface high‐entropy sulfide (CdMnCoZnNi)S_x_ as the photoactive material and a PtRuFeCoMn HEAzyme as a signal amplifier, resulting in an LOD of 0.75 pg mL^−1^ [326]. Another PEC sensor, designed for patulin detection, utilized nanotube‐like In_2_S_3_/WO_3_ heterostructures and PtRuFeCoW HEA nanorods as a catalytic amplifier [327]. This work showing the detection limit as low as 0.063 pg mL^−1^. Additionally, a smartphone‐assisted colorimetric paper sensor based on CuZnCoNiFe HEA was developed for rapid assessment of antioxidant levels, enabling the distinction between naturally and artificially ripened fruits for food freshness evaluation [328]. The robust POD‐like activity of HEAzymes has been harnessed for the detection of biothiols and related enzyme activities. Zhang et al. integrated FePtCoNiCu HEAzymes into a hydrogel matrix, establishing a portable, cost‐effective colorimetric platform [329]. This system enabled the rapid and sensitive detection of biothiols, as well as the monitoring of acetylcholinesterase activity and organophosphorus pesticides, facilitated by smartphone‐based imaging for on‐site analysis. As shown in Figure 14C, a high‐ entropy Prussian blue analogue oxide (MnCoNiCuZnFe) with multienzymatic activity was employed for glutathione detection [330]. The colorimetric system, combined with a microfluidic paper‐based analytical device, demonstrated low detection limits, excellent selectivity, and long‐term stability, with DFT calculations revealing the Gibbs free energy of H_2_O_2_ dissociation on individual transition‐metal sites.

Beyond specific analyte detection, several studies have focused on developing novel strategies and providing mechanistic insights into HEAzymes [331]. The concept of HEAzymes was demonstrated using PdMoPtCoNi HEA nanowires, which exhibited POD‐mimicking activity comparable to that of natural horseradish POD (Figure 14D) [332]. DFT calculations attributed this activity to enhanced electron abundance near the Fermi level via a self‐complementation effect. A novel signal amplification strategy based on controlled nanozyme‐substrate accessibility was introduced by Huang et al., in which a ferrocene/Au/DNA composite acted as a signal‐shielding layer, enabling highly sensitive and specific miRNA detection with an LOD of 1.67 fm [333]. Surface engineering was also explored as a means to optimize catalytic activity. For instance, PtRuFeCoNi HEA nanowires modified with polydopamine (HEAzymes@PDA) showed enhanced adsorption‐desorption efficiency due to a moderately lowered d‐band center, consistent with the Sabatier principle [209]. In a high‐throughput approach, a library of 81 multimetallic oxide‐based nanozymes was synthesized to investigate the relationship between compositional complexity and catalytic performance [334]. A generalized linear model was developed, identifying synergistic and antagonistic elemental interactions and introducing the mean interaction effect as a valuable parameter for the rational design of HEAzymes.

### 4.4 HEAzyme toward for Future Energy Catalysis

Beyond their established roles in biosensing, HEAzymes are emerging as versatile platforms for energy conversion, environmental remediation, and advanced manufacturing [335, 336, 337]. By leveraging multielement synergy and configurational entropy stabilization, these materials overcome the single‐functionality limitations of conventional nanozymes, enabling multimodal catalysis while maintaining exceptional structural integrity under extreme conditions [338]. This section surveys recent advances in these emerging domains, from marine protection to electrochemical energy systems. Antifouling and environmental biotechnology. Conventional antifouling coatings rely heavily on toxic biocides that threaten marine ecosystems. In response, a bio‐inspired HEOs, Ce_0.2_Zr_0.2_Nb_0.2_Ta_0.2_Hf_0.2_O_x_, has been developed as a biocide‐free coating with intrinsic enzyme‐mimetic properties [339]. The lattice simultaneously simulates lactonase, protease, and haloperoxidase functions, hydrolyzing adhesive proteins and quorum‐sensing molecules to prevent biofilm formation without toxic leaching. DFT calculations attribute this interfacial reactivity to the Ce d‐band center near the Fermi level, which facilitates efficient electron transfer. Prolonged ocean‐side immersion trials confirm that such single‐layer coatings effectively resist both macro‐ and micro‐fouling, establishing an environmentally benign paradigm for long‐term underwater protection [340]. It is worth noting that HECs have also been explored in energy conversion and storage systems, such as electrocatalytic water splitting, rechargeable batteries, and photothermal evaporation [341, 342, 343]. A high‐entropy single‐atom metal‐doped porous carbon with an ultralow metal loading of 1.77 wt% was designed as a photothermal converter featuring tunable hierarchical porosity [344]. When integrated into a cone‐shaped evaporator with optimized 3D light harvesting, the system achieves 2.86 kg m^−2^ h^−1^ water evaporation rate and 94.5% solar‐to‐vapor efficiency. Molecular dynamics simulations reveal that this architecture significantly elevates intermediate water content and accelerates water transfer kinetics.

While HEMs have previously been explored in classical electrocatalysis and photothermal systems, those early investigations typically operated through conventional electrochemical mechanisms without exhibiting enzyme‐like Michaelis–Menten kinetics. Nevertheless, the structural insights gained from those studies provide a transferable foundation for true HEAzyme‐based energy catalysis. Oxygen evolution reaction. In anodic water splitting, high‐entropy metal hydroxide organic frameworks synthesized under mild solvothermal conditions construct hydroxide layers with randomly distributed cations that form synergistic catalytic networks (Figure 15A) [345]. The material requires only ≈1.64 V to reach 100 mA cm^−2^ and maintains stable operation for over 100 h at 10 mA cm^−2^, outperforming precious metal benchmarks. First‐principles calculations and operando X‐ray absorption spectroscopy reveal that the high‐entropy configuration induces a multifaceted OER mechanism, effectively reducing reliance on high loadings of active metal while eliminating noble metals entirely. Atomically dispersed PtRhBiSnSb high‐entropy intermetallic nanosheets demonstrate precise regulation of multimetallic synergy at the atomic scale (Figure 15B) [346]. Within their hexagonal close‐packed lattice, the isolated distribution of five metals endows the catalyst with outstanding multifunctional alcohol oxidation activity, achieving record mass activities of 19.529, 15.558, and 7.535 A mg^−1^ Pt+Rh for methanol, ethanol, and glycerol, respectively, in alkaline media. Theoretical calculations indicate that Rh enhances electron transfer efficiency, while Bi, Sn, and Sb sites synergistically stabilize the electronic structure of active centers‐highlighting how high‐entropy design can tame complex multi‐electron reactions. These findings demonstrate the promise of HEMs in advancing solar thermal management technologies. In parallel, HEAzymes are gaining increasing attention in the field of energy conversion and storage, where their multielement synergistic effects and tunable electronic structures offer new pathways for addressing key scientific challenges [347]. As shown in Figure 15C, on the cathodic side, non‐precious‐metal HEA nanoflower arrays have enabled industrial‐scale hydrazine oxidation‐assisted hydrogen production [348]. Benefiting from a single‐phase solid‐solution structure, these electrodes drive current densities from 10 to 2000 mA cm^−^^2^ at ultralow voltages while maintaining 300 h of stable operation. The performance stems from an intelligent migration of key HzOR intermediates that dynamically optimizes the reaction pathway and continuously regenerates high‐activity sites. In parallel, as show in Figure 15D, size‐controlled PdPtRuRhAu alloys achieve 100 h of stable acidic HER. The high proportion of Pd–Au bridge adsorption sites serve as the structural origin of this intrinsic activity (Figure 15D) [349]. Notably, machine learning model points accurately predicts the ability and establishing a data‐driven paradigm for HEAzyme design.

**FIGURE 15:**
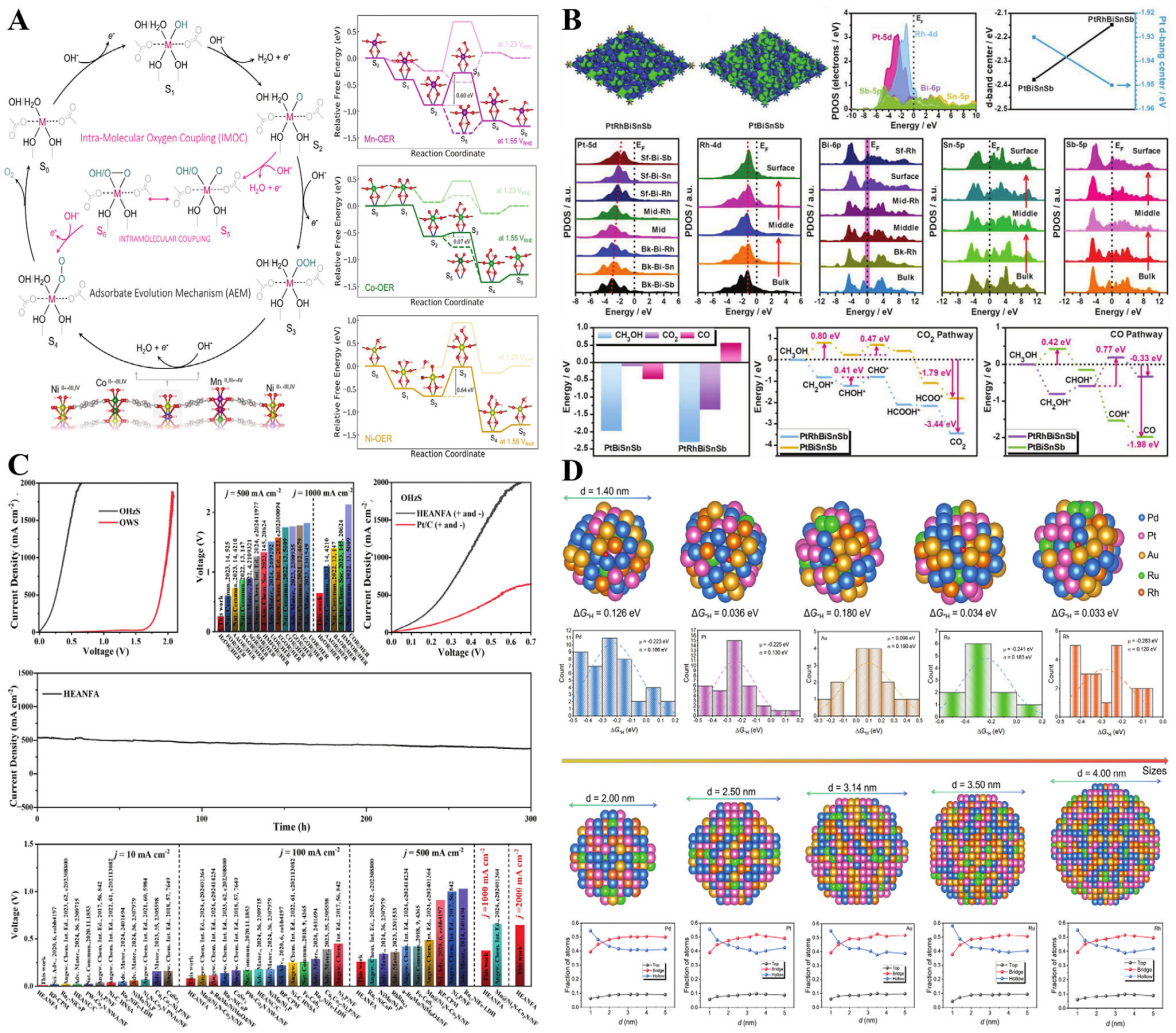
HEAzymes for energy catalysis: (A) High‐entropy metal hydroxide organic framework for oxygen evolution reaction. Reprinted with permission from Ref. [345] Copyright 2024, Wiley‐VCH. (B) High‐entropy PtRhBiSnSb for alcohol oxidation electrocatalysis. Reprinted with permission from Ref. [346] Copyright 2022, Wiley‐VCH. (C) HEAs for low‐voltage hydrogen production. Reprinted with permission from Ref. [348] Copyright 2025, Wiley‐VCH. (D) Size‐controllable HEAs for stable hydrogen production. Reprinted with permission from Ref. [349] Copyright 2025, Wiley‐VCH.

Entropy‐stabilization strategies are also advancing electrolyte engineering [350, 351, 352]. For potassium‐ion batteries, an entropy‐repair concept was introduced to re‐engineer the K^+^ solvation shell, yielding a high‐entropy localized high‐concentration electrolyte that delivers a steady coulombic efficiency of 99.4% and stable cycling for over 10 000 h under high current density. This illustrates that entropy engineering extends far beyond catalysis to stabilize electrochemical interfaces in rechargeable systems [353]. These advances demonstrate that HEAzymes unify high activity, poisoning resistance, and long‐term durability across OER, alcohol oxidation, and HER through configurational‐entropy‐driven multimetallic synergy, dynamic active‐site optimization, and reaction‐pathway re‐engineering. By substantially reducing dependence on scarce noble metals while matching or exceeding benchmark performance, HEAzymes offer a sustainable pathway for next‐generation fuel cells, metal‐air batteries, and industrial hydrogen production. As precise synthesis, operando characterization, and machine learning‐guided design continue to converge, HEAzymes are positioned to play an increasingly transformative role in the global transition toward clean and efficient energy technologies [354, 355, 356].

## 5 Summary and Outlook

In conclusion, this review has systematically examined the development of HEAzymes, encompassing synthetic methodologies, catalytic mechanisms, and application landscapes. We have categorized synthesis strategies into thermodynamically driven and kinetically controlled approaches, while elucidating the structure‐activity relationships underlying their multienzyme catalytic capabilities. The significant progress achieved in therapeutic applications, biosensing platforms, and energy conversion systems demonstrates the potential of HEAzymes to transcend the limitations of conventional nanozymes and establish new paradigms in bioinspired catalysis. Despite the rapid progress in HEAzyme research, the field remains confronted with a constellation of interdependent challenges that span from fundamental understanding to practical deployment. These bottlenecks are not isolated; rather, they form an interconnected hierarchy in which limitations at one level propagate and amplify obstacles at the next. A systematic dissection of these barriers across four critical dimensions of fundamental research, fabrication, characterization, and practical application are essential to chart a viable path toward rational design and clinical translation.

### 5.1 Fundamental‐Research Problems

At the foundational level, HEAzymes lack the atomic‐scale understanding necessary for true rational design. The precise 3D architecture of catalytic active sites remains experimentally unresolved, leaving a critical mechanistic void: it remains unclear whether enhanced catalytic performance stems from d‐band‐center modulation, lattice‐distortion/strain effects, multi active site synergy, or merely the superposition of individual elemental contributions. Notably, the widely invoked “cocktail effect” has yet to be rigorously validated as an authentic catalytic mechanism. In the absence of such mechanistic clarity, structure activity relationships remain largely correlative rather than predictive, precluding knowledge‐driven optimization. Compounding this issue, the catalytic activity and substrate selectivity of existing HEAzymes still lag orders of magnitude behind those of natural enzymes, severely constraining their utility in precision biomedicine. Moreover, the mechanisms by which high‐entropy configurations stabilize specific oxidation states or coordination environments during catalytic turnover remain poorly understood, rendering current design strategies reliant on empirical exploration rather than first‐principles guidance.

### 5.2 Fabrication‐Related Obstacles

These fundamental knowledge gaps directly translate into severe fabrication bottlenecks. Without predictive structure‐activity models, synthesis protocols remain largely empirical, offering limited control over nucleation and growth kinetics. Conventional methods such as hydrothermal and sol‐gel approaches are particularly vulnerable during scale‐up, where minute fluctuations in temperature, pH, or precursor mixing rates readily induce elemental segregation, broadened size distributions, and morphological heterogeneity. Consequently, batch‐to‐batch reproducibility remains poor, and the inherent polydispersity of current products obscures intrinsic structure‐property correlations. Achieving atomic‐level compositional homogeneity is extraordinarily challenging, as differential reduction potentials and incompatible ionic radii among constituent metals drive thermodynamically favored phase separation. Although emerging techniques such as atomic layer deposition and microfluidics‐assisted synthesis offer enhanced precision, they currently suffer from low throughput, technical complexity, and incompatibility with the gram‐scale production required for preclinical evaluation. In the absence of robust, scalable protocols that guarantee compositional and structural fidelity, systematic benchmarking across independent research groups remains effectively unattainable.

### 5.3 Characterization‐Technique Limitations

Compounding these fabrication challenges are profound limitations in characterization methodologies. Even when relatively homogeneous samples are obtained, the analytical tools available to probe HEAzyme structure and function under operationally relevant conditions remain inadequate. The majority of structural insights currently derive from ex situ, static measurements that capture only post‐reaction snapshots, thereby missing the dynamic evolution of active centers during catalytic turnover. Advanced operando and in situ techniques of synchrotron radiation X‐ray absorption spectroscopy, environmental transmission electron microscopy, and Raman spectroscopy coupled with electrochemical cells are technically demanding, susceptible to beam damage, and frequently incompatible with the liquid‐phase environments central to biomedicine. Furthermore, the complex, multielement nature of HEAzymes generates heavily convoluted spectroscopic signatures that are difficult to deconvolute without machine‐learning‐assisted analysis; however, curated training datasets for such algorithms remain scarce. The simultaneous temporal and spatial resolution required to track atomic rearrangements at active sites during enzymatic mimicry is still beyond the reach of current instrumentation, leaving the critical link between local atomic environment and catalytic function largely inferred rather than experimentally validated.

### 5.4 Practical‐Application Barriers

The confluence of unresolved mechanistic questions, fabrication inconsistencies, and characterization deficiencies collectively erects formidable barriers to practical application. Even assuming that synthetic and analytical hurdles can be surmounted, the pathway to clinical and industrial deployment remains obstructed by unresolved translational issues. Long‐term biosafety profiles, including chronic cytotoxicity, immunogenicity, organ accumulation, and metabolic fate are yet to be systematically evaluated in physiologically relevant microenvironments. Existing studies rely predominantly on simplistic 2D cell assays, whereas more sophisticated models such as organoids, organ‐on‐a‐chip platforms, and 3D tissue constructs remain underutilized. From a regulatory perspective, the absence of standardized activity measurement protocols, certified reference materials, and unified evaluation frameworks renders cross‐study comparison impossible and impedes regulatory approval. In therapeutic contexts, the limited repertoire of mimicked enzyme activities, insufficient catalytic specificity, and lack of active targeting functionalities restrict HEAzymes to broad, non‐specific applications rather than enabling precision interventions. Additionally, critical practical considerations, such as integration with existing manufacturing workflows, sterilization compatibility, and shelf‐life stability under clinical storage conditions remain entirely unaddressed. Until these translational bottlenecks are systematically mapped and resolved, HEAzymes risk remaining confined to the laboratory as promising conceptual constructs rather than realizing their potential as practical technological solutions.

### 5.5 Integrated Path Forward

Addressing this interconnected array of challenges necessitates a coordinated, multidisciplinary paradigm shift. To bridge the fundamental knowledge gap, rational design must be empowered by the integration of artificial intelligence and high‐throughput virtual screening, leveraging machine‐learning algorithms trained on standardized experimental datasets to navigate the vast compositional space of multimetallic systems and predict optimal elemental combinations and atomic configurations. On the fabrication front, synthesis workflows should evolve toward automated, closed‐loop systems that couple robotic material fabrication with real‐time characterization feedback, thereby ensuring compositional fidelity and reproducibility. Mechanistic understanding, in turn, demands the widespread adoption of operando characterization methods combined with advanced data analytics to unravel entropy‐driven stabilization effects and authentic synergistic catalytic mechanisms. Finally, performance evaluation must transition from simplistic activity assays toward multidimensional functional assessments employing cutting‐edge biological models, thereby bridging the persistent divide between in vitro discovery and in vivo translation. Ultimately, the deep integration of materials science, catalysis, computational chemistry, biology, and clinical medicine will be indispensable to transform HEAzymes from an emerging conceptual advance into a robust, next‐generation nanozyme platform.

## Author Contributions

**Yongjian Ai**: conceptualization, investigation, funding acquisition, writing – original draft, writing – review and editing. **Weibo Zhang**: methodology, data curation, writing – original draft. **Pengjie Wang**: data curation, software, funding acquisition. **Wentao Xu**: conceptualization, supervision, data curation. **Huiyuan Guo**: conceptualization, data curation, supervision, writing – review and editing. **Hong‐bin Sun**: writing – review and editing, data curation, supervision. **Meng‐Qi He**: supervision, data curation, writing – original draft, writing – review and editing, conceptualization. **Fazheng Ren**: conceptualization, writing – review and editing, funding acquisition, data curation, supervision, resources. **Qionglin Liang**: resources, supervision, data curation, writing – review and editing, funding acquisition.

## Funding

This work was financially supported by the Beijing Nova Program (202604841266), National Natural Science Foundation of China (82574366, 22304099, 82304442), National Key R&D Program of China (2023YFC3504401, 2022YFA1103403), Key Special Project of the Science and Technology Revitalizing Inner Mongolia Action (NMKJXM202204), Beijing Natural Science Foundation (L256002) and Chinese Academy of Medical Sciences Innovation Fund for Medical Sciences (2024‐I2M‐3‐013, 2023‐I2M‐2‐001, 2026‐I2MSXZX4‐06).

## Conflicts of Interest

The authors declare no conflicts of interest.

## Data Availability

The data that support the findings of this study are available from the corresponding author upon reasonable request.
